# RNA editing by ADAR1 leads to context-dependent transcriptome-wide changes in RNA secondary structure

**DOI:** 10.1038/s41467-017-01458-8

**Published:** 2017-11-13

**Authors:** Oz Solomon, Ayelet Di Segni, Karen Cesarkas, Hagit T. Porath, Victoria Marcu-Malina, Orel Mizrahi, Noam Stern-Ginossar, Nitzan Kol, Sarit Farage-Barhom, Efrat Glick-Saar, Yaniv Lerenthal, Erez Y. Levanon, Ninette Amariglio, Ron Unger, Itamar Goldstein, Eran Eyal, Gidi Rechavi

**Affiliations:** 1 Cancer Research Center, Sheba Medical Center, Tel Hashomer, 52621 Ramat-Gan, Israel; 2 The Wohl Institute for Translational Medicine, Sheba Medical Center, Tel Hashomer, 52621 Ramat-Gan, Israel; 3 The Mina and Everard Goodman Faculty of Life Sciences, Bar-Ilan University, 52900 Ramat-Gan, Israel; 4 Department of Molecular Genetics, Weizmann Institute of Science, 76100 Rehovot, Israel; 5 Sackler School of Medicine, Tel Aviv University, 69978 Tel-Aviv, Israel

## Abstract

Adenosine deaminase acting on RNA 1 (ADAR1) is the master RNA editor, catalyzing the deamination of adenosine to inosine. RNA editing is vital for preventing abnormal activation of cytosolic nucleic acid sensing pathways by self-double-stranded RNAs. Here we determine, by parallel analysis of RNA secondary structure sequencing (PARS-seq), the global RNA secondary structure changes in ADAR1 deficient cells. Surprisingly, ADAR1 silencing resulted in a lower global double-stranded to single-stranded RNA ratio, suggesting that A-to-I editing can stabilize a large subset of imperfect RNA duplexes. The duplexes destabilized by editing are composed of vastly complementary inverted Alus found in untranslated regions of genes performing vital biological processes, including housekeeping functions and type-I interferon responses. They are predominantly cytoplasmic and generally demonstrate higher ribosomal occupancy. Our findings imply that the editing effect on RNA secondary structure is context dependent and underline the intricate regulatory role of ADAR1 on global RNA secondary structure.

## Abstract (web summary)

Adenosine deaminase acting on RNA 1 (ADAR1) edits adenosine to inosine. Here the authors, using parallel analysis of RNA secondary structure sequencing, provide evidence that ADAR1 induces sequence-context-dependent RNA secondary structures changes, often leading to stabilization of the RNA duplex.

## Introduction

Deamination of adenosine to inosine, A-to-I RNA editing^1–5^, is the most widespread RNA-editing type in humans. This modification is catalyzed by the ADAR (adenosine deaminases acting on RNA) protein family. Most editing events are mediated by ADAR1 (gene symbol—*ADAR*) with two distinct protein isoforms p110 and p150. Double-stranded RNA (dsRNA), often formed between adjacent inverted repetitive (IR) elements, is the obligatory substrate of ADAR. Thus, a large fraction of editing sites occurs in Alu retroelements, interspersed within non-coding regions of mRNA transcripts including introns and untranslated regions (UTRs). The high abundance of the primate-specific Alu retroelements in the human genome, is a major cause of most of the RNA-editing activity in the human transcriptome^6, 7^. There are millions of editing sites in non-coding regions and although many are edited at only a tiny fraction of the transcripts, there are still thousands of sites where the editing level is significant^8^, suggesting a functional role.

ADAR1 is required for normal fetal development. Knocked out mice die at an early embryonic stage (day 11.5) with defects in hematopoiesis and liver disintegration^9, 10^ apparently due to abnormal induction of a type-I interferon response. In hematopoietic stem cells, loss of ADAR1 led to global up regulation of interferon-stimulated genes and apoptosis^11^. ADAR1 is also vital for post-natal hepatic development and homeostasis, with hepatocyte-specific depletion of ADAR1 leads to liver inflammation and necrosis^12, 13^. Likewise, ADAR1 is vital for B lineage development^14^.

dsRNA is a major inducer of anti-viral type-I interferon response via activation of cytosolic nucleic acid sensing pathways^15^. An essential role of A-to-I RNA editing is inhibition of the immune response to perfect self-dsRNA by editing such regions.^16,17–19^. Indeed, bi-allelic mutations in ADAR1 were documented to cause Aicardi–Goutieres syndrome, an inflammatory genetic syndrome associated with toxic perturbation of the type-I interferon response (“interferonopathy”)^20^. Recent studies directly connect the severe phenotypes of ADAR1 deficiency to its mRNA editing activity^21^. ADAR1 is assumed to prevent activation of relevant cytosolic sensors of nucleic acids (mainly MDA5) by RNA duplexes dispersed in the human transcriptome.

Editing may regulate RNA abundance and fate by changing the local structure^22^. This may change the stability of the RNA duplex, affect structural determinants recognized by trans-acting factors and can change accessibility of factors to defined regions^23–25^. As even a single-nucleotide variant may affect RNA structure^26–28^, RNA editing may well affect the transcriptome secondary structure landscape. Furthermore, A-to-I editing events frequently occur in clusters (hyper-edited regions), and such regions may undergo major structural alterations. Indeed, early pioneering studies demonstrated an unwinding activity for ADAR enzymes^29, 30^.

Despite the accumulating evidence regarding the regulation of transcript structures by ADAR1 and the role of this regulation in health and disease, no study investigated the structural changes induced by ADAR using an experimental global approach.

Recently, new methodologies were developed for experimental identification of RNA 2D structure in a transcriptome wide manner using next generation sequencing (NGS). Some are based on probing with single-strand (SS) and double-strand (DS) specific nucleases^31, 32^. Using such approaches, the global yeast, thale cress, fruit fly, nematode and human transcriptome 2D structures, were mapped^27, 33–35^. Methods that are based on chemical probing^36^, more suitable for in vivo studies, were also developed and applied^37–40^.

Here, we report the use of high-throughput probing techniques to directly examine, the effect of ADAR1 deficiency and A-to-I editing on the whole transcriptome 2D structure and relate the data to the regulation of the cytosolic innate immune response. We show that in human cells, hundreds of transcripts undergo ADAR1 editing-dependent structural changes. In contrast to the prevailing paradigm that ADAR1 activity decreases RNA duplex stability, we find that A-to-I editing may increase the stability of RNA duplexes and that the effect on the global RNA structure is context dependent. The smaller subset of genes whose transcripts are destabilized upon editing, as previously postulated, are significantly enriched for housekeeping genes and genes linked to the anti-viral interferon response. These transcripts tend to be localized in the cytoplasm, and are relevant to the involvement of ADAR1 in dampening innate immune response.

## Results

### PARS-seq of HepG2 cells captures RNA 2D structure

**Fig. 1:**
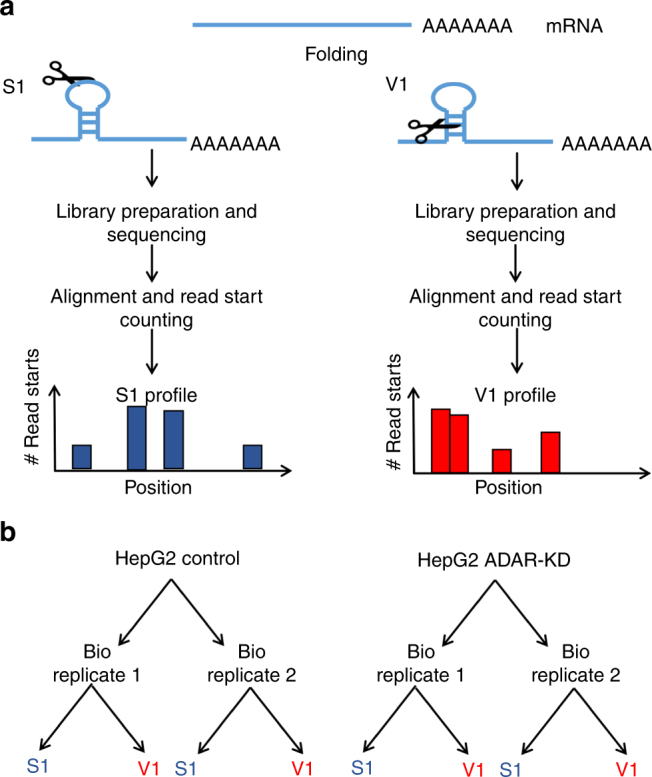
PARS-seq experimental flow. **a** Construction of single-stranded (SS) or double-stranded (DS) RNA profile is done by digestion with S1 or V1, respectively. **b** Samples used in this study

In order to examine global RNA structural changes induced by ADAR1 silencing, we applied PARS-seq^32^ as detailed in the Methods section. Purified poly-A + RNA samples from control HepG2 cells and ADAR knockdown (KD) HepG2 cells were extracted and in vitro probed with S1 nuclease (S1) and RNAse V1 (V1) that preferentially cleave RNA at SS and DS regions, respectively (Fig. 1 and Supplementary Table 1). The RNA was then reverse-transcribed and sequenced using Illumina HiSeq 2500. To uncover S1 or V1 digestion profiles we counted the number of reads that start at each base of the analyzed RNA molecule. The procedure was performed in two biological replicates for each treatment (Fig. 1).

**Fig. 2:**
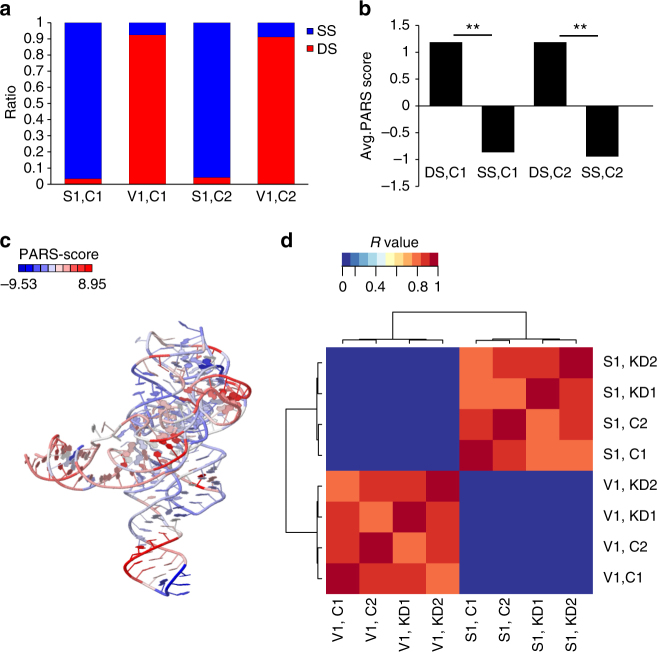
Verification of the PARS-seq 2D structural data. **a** Agreement with solved RNA structure. Ratio between the sum of read starts at known SS or DS bases based on the solved structure of *Tetrahymena* ribozyme (PDB ID: 1x8w). Blue: bases known as SS bases. Red: bases known as DS bases. S1, C1: control 1 cleaved by S1. V1, C1: control 1 cleaved by V1. S1, C2: control 2 cleaved by S1. V1, C2: control 2 cleaved by V1. **b** Mean PARS score. SS, C1: bases known as SS in control 1. DS, C1: bases known as DS in control 1. SS, C2: bases known as SS in control 2. DS, C2: bases known as DS in control 2. **Wilcoxon test, *p* < 0.01 **c** The solved structure of p4–p6 domain of the *Tetrahymena* ribozyme (PDB ID: 1x8w) colored by the PARS scores obtained in this study. Blue: PARS score < 0 (SS region). Red: PARS score > 0 (DS region). **d** Spearman’s correlations were calculated between vectors per sample, where each vector element contains the number of reads which start at a particular base (correlation here is at single-nucleotide resolution). The correlation between samples is based on the 1000 most highly expressed genes to ensure sufficient coverage at the base level. Red: Spearman’s *R* = 1. Blue: Spearman’s *R* = 0. As expected, correlations between V1 and S1 samples are low, further confirming the PARS-seq procedure

To verify that the PARS-seq protocol reliably deciphers the structural profiles of the analyzed RNA molecules, we compared the structural profile obtained for a known control, the p4–p6 domain of the *Tetrahymena* ribozyme, inserted together with the studied human RNA, to its previously determined structure (PDB ID: 1x8w). We show that the mean PARS score is higher in known DS bases compared to known SS bases (Fig. 2b). The sum of raw V1 read starts is higher in DS bases compared to known SS bases while a reverse trend exists for S1 read starts (Fig. 2a), implying that the observed structural profile matches the solved 3D structure (~90% of the reads agree with the known structure, Fig. 2b, c). We also examined natively expressed human RNA molecules with a known fold. The PARS-seq structural profile of the human tRNA-ARG (UCSC ID: uc021ucc) matches the solved RNA structure of its yeast homolog (PDB ID: 1f7u, about ~60–80% of the reads agree, Supplementary Fig. 1) and the structural profile of human U2 (UCSC ID: uc021xtz) matches its conserved structure (Supplementary Table 2; Rfam ID: RF00004). Additionally, we verified that our PARS scores for long RNA molecules, such as the long non-coding RNA (lncRNA) MALAT1, show significant correlation between biological replicates (single-nucleotide resolution, Spearman’s correlation of *R* = 0.94). We also verified that calculated PARS scores from a previous study of lymphoblastoid cells transcriptome^27^ significantly correlate with our scores (Spearman’s *R* = 0.6), indicating that the PARS-seq approach we utilize reliably captures the global structural features of native long RNA molecules in different cell types. The V1 profiles obtained are significantly different from the S1 profiles in all the samples while the profiles of replicates are nicely correlated (heat maps in Fig. 2d and Supplementary Fig. 1d).

In agreement with a previous study that applied a similar methodology^32^, start and stop codons indeed showed local minima of PARS score with major differences found in 2D structure near translation start/stop sites (Supplementary Fig. 2a and Supplementary Table 3). We likewise observed 2D structure periodicity at the CDS with lower PARS scores at the first base of the codons (Supplementary Fig. 2b, c and Supplementary Table 4). A significant trend (Wilcoxon test, *p* < 0.005) was detected for excess of V1 reads (DS) within Alu retroelements (Supplementary Table 5) as Alu elements in inverted orientation (IR) result in DS structures. Some specific RNA sites are not represented in neither the V1 nor the S1 libraries in PARS-seq experiments. Some transcripts expressed at low level may escape detection due to inadequate coverage. We considered an additional less trivial explanation for lack of detection, assuming that some sequences are not accessible to the solvent in the 3D fold and therefore cannot be digested by both V1 and S1 enzymes^41^. We put this hypothesis to the test and analyzed the *Tetrahymena* ribozyme domain p4–p6 that was used as control and checked if minimally cleaved regions have low solvent accessibility as computed based on the solved 3D structure (PDB ID: 1x8w). Indeed, non-accessible (buried) bases were found to have, on average, lower read coverage in both V1 and S1 libraries (*p* = 0.02, Supplementary Table 6).

### Effects of ADAR1 silencing on editing and gene expression

We silenced ADAR1 in HepG2 cells as described in the methods section. In brief, we used validated siRNA for ADAR1 (siADAR1) and scrambled siRNA to generate ADAR1 knockdown (KD) and control cells, respectively (Methods). quantitative reverse transcription (qRT)-PCR and western-blotting (Supplementary Fig. 3) showed an effective ADAR1 KD of ~90%. RNA-seq and PARS-seq verified this observation with 72 and 66% reduction of ADAR1 transcripts, respectively. 131,086 known potential editing sites (from the A-to-I RNA-editing collections in DARNED^42^ and RADAR^8^) were found to be edited in at least one PARS-seq sample. As expected, significant global decrease in RNA-editing level was observed in ADAR1 deficient cells (Supplementary Fig. 4a; *p* < 10^−15^). Moreover, editing sites located in IR Alus in 3′UTR of genes, which are predominantly ADAR1 targets, showed reduction of about 70% in editing level (Supplementary Fig. 4f; *p* < 10^−15^). To further identify sequence reads that are edited at many positions and could not be mapped by standard NGS alignment tools that were used to detect the known 131,086 editing sites, we also applied de novo detection of hyper-edited sites as described in Porath et al.^43^ Both numbers and levels of editing sites were reduced also in this set in the ADAR1 deficient cells (Supplementary Fig. 4b, e). A total of 5917 sites from this analysis were more confidently called, as they are located within uniquely aligned reads, and thus were added to the previous 131,086 set for further analysis.

The significant enrichment (Supplementary Fig. 4c) of A-to-G mismatches compared with all other mismatches proves that our methodology is both sensitive and specific for A-to-I editing detection as previously described^3^. A strong ADAR1 binding motif was identified in the newly identified edited sites (using the Porath et al.^43^ pipeline), as demonstrated by under-representation of G at (−1) to the editing site (Supplementary Fig. 4d). Overall, 137,003 editing sites were identified in at least one sample and were further intersected with the structural results. Complete lists of the editing sites in this study are available in Supplementary Data 1.

We identified 5644 RNA-editing sites which have significantly lower editing level in ADAR1 KD than in control cells (*p* < 0.05). 508 of the sites also passed false discovery rate (FDR) filter (*q* < 0.1) in the S1 samples or in the V1 samples. These differentially edited sites (DES) are likely specific targets of ADAR1 (Supplementary Data 2). DES have the potential to directly change Watson–Crick interfaces in the RNA, or alter binding sites of RNA-binding proteins that help to stabilize RNA in particular conformations.

The expression of ADAR2 (synonym: *ADARB1*) was increased by ~2.2 fold in ADAR1 KD cells (FDR < 0.05 in both V1 and S1 samples), in line with the previous demonstration of a reciprocal compensatory change in abundance/activity between ADAR2 and ADAR1^23, 44^. Editing sites that demonstrate lower editing level in KD samples are therefore most likely ADAR1 substrates (DES, Supplementary Data 2), while sites with higher editing in KD are probably the targets of ADAR2 (only 174 sites, included in Supplementary Data 3). The high ratio of ADAR1 to ADAR2-editing targets found here (~32-fold), further verifying our approach.

### Regions with ADAR1-regulated secondary structure changes

To identify transcripts where the V1 or S1 profiles were significantly changed between control and ADAR1 KD, we compared the correlation between the vectors of read start counts for each RefSeq gene. We derived the correlation difference (CorDiff) as an objective score for this analysis and filtered the data to retain only those transcripts with CorDiff > 0 in both V1 and S1 samples, that conform with a random shuffling test to avoid low sampling bias (Methods). 3196 RefSeq transcripts with secondary structure changes (SSC) had CorDiff > 0 in both V1 and S1. In total, 2155 of these transcripts have original CorDiff that is higher than 95% of the random trials (empirical *p* < 0.05, Supplementary Data 4). Similar analysis was also performed for individual exons (Supplementary Datas 5–6). Analysis using DAVID^45^ and GOrilla^46^ of SSC transcripts revealed significant enrichment of biological and molecular functions related to ribonucleotide binding, mitosis, nuclear division, ATPase activity, RNA binding and p53 binding (FDR < 0.05; Supplementary Data 7). We found that SSC in 3′UTRs were enriched for DES (Table 1) and for IR Alu retroelements separated by <4000 bases^3^ (Supplementary Table 7), as compared with a control set of 3′UTRs with similar coverage that were not altered. In addition, we found that the SSC 3′UTR exons overlapped with ADAR1 binding sites previously identified by Clip-seq (Supplementary Table 7)^47^. The lists of RNA-editing sites and DES in SSC 3′UTR exons are available in Supplementary Datas 8–9.

**Table 1:** RNA editing is enriched within structurally changed (SSC) 3′UTR exons

	# RNA-editing sites^a^	Total genomic size (# bases)	Fold	*p*-value
SSC 3′UTR (avg. read starts > 50^b^)	1479	2,274,485	1.38	<10^−15^
Non-SSC 3′UTR (avg. read starts > 50)	9157	19,443,239		
SCC 3′UTR (avg. read starts > 200)	1293	1,550,212	1.65	<10^−15^
Non-SSC 3′UTR (avg. read starts > 200)	8227	16,311,452		
SSC 3′UTR (avg. read starts > 1000)	618	433,095	2.51	<10^−15^
Non-SSC 3′UTR (avg. read starts > 1000)	5303	9,328,336		
	*# DES* ^c^	*Total genomic size (# bases)*	*Fold*	*p-value*
SSC 3′UTR (avg. read starts > 50)	213	2,274,485	1.5	5.93 × 10^−10^
Non-SSC 3′UTR (avg. read starts > 50)	1215	19,443,239		
SCC 3′UTR (avg. read starts > 200)	206	1,550,212	1.88	<10^−15^
Non-SSC 3′UTR (avg. read starts > 200)	1152	16,311,452		
SSC 3′UTR (avg. read starts > 1000)	121	433,095	2.93	<10^−15^
Non-SSC 3′UTR (avg. read starts > 1000)	891	9,328,336		

^a^Known RNA-editing sites and de novo detected hyper-edited sites (detected by the pipeline of Porath et al.^43^) which are edited in the PARS-seq samples

^b^Averaged number of read starts in all the PARS-seq samples

^c^RNA-editing sites that showed significantly lower editing levels G/(A + G) in ADAR KD samples

The observed structural changes may affect cellular physiology via several mechanisms. Structural alterations may affect binding specificity and avidity of RNA-binding proteins or ncRNAs. Search for SSC in 3′UTRs with long IR Alu regions revealed that these regions are enriched with binding sites for STAU1, an RNA binding protein which binds DS RNA and activates Staufen-mediated-decay (Supplementary Table 8, based on STAU1 hiCLIP data; Methods section)^48^. IR Alus in 3′UTRs that are influenced by editing-mediated SSC are also enriched by miR binding sites (Supplementary Table 9). Additional possible molecular outcomes of editing-mediated SSC include alteration of binding of lncRNAs, of splicing or 3′UTR shortening and change in nuclear/cytoplasmic mRNA localization and stability^22^.

We showed before that ADAR indeed participates in alternative splicing regulation^49^. Since alternative splicing might be interpreted as SSC, we looked at single isoform genes, where there is no splicing that can affect transcript secondary structure. We found a similar (6.4%) fraction of SSC exons in single isoform genes compared to non-SSC exons (Supplementary Table 10), suggesting that alternative splicing does not play a significant role in the structural changes observed upon ADAR silencing. We also performed independent analysis to detect alternatively spliced exons based on the PARS-seq data using DEXSeq^50^ (Supplementary Data 10) and found that only ~22% of the SSC exons are identified as alternatively spliced (Supplementary Table 11). These findings indicate that most of the observed differences actually reflect local structural changes.

### Structural changes in 3′UTRs of selected genes

**Fig. 3:**
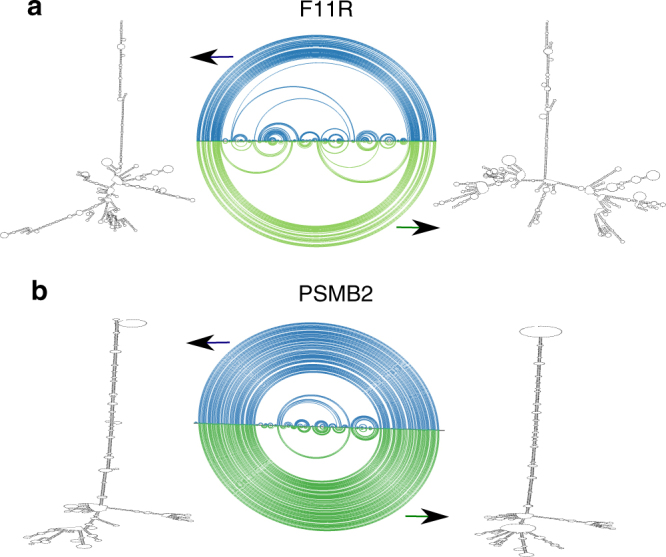
Examples of SSC in hyper-edited 3′UTRs. The arc diagrams show the base pairing in IR Alus in control (blue) and KD (green). **a** Prediction of control and KD structures using the PARS-seq data of F11R SSC 3′UTR IR Alu region (chr1:160966275-160967962/hg19) as constrains for RNAfold. Corresponding 2D structure diagrams from control (left) and KD (right) samples are shown. **b** Prediction of control and KD structures using the PARS-seq signals of PSMB2 IR Alu region (chr1:36066658-36067955/hg19) as constrains for RNAfold. Corresponding 2D structure diagrams of control (left) and KD (right) are shown. The change in base pairing is represented in both examples by the differences in the arcs in control and KD samples

SSC 3′UTR are enriched with editing sites and DES (see above). Illustrative example for such cases, reflecting changes in mRNA folding are shown in Fig. 3a. The 3′UTR of F11R is highly edited with 24 editing sites identified; at least 5 of them are DES (for the full DES list see Supplementary Data 2). A major change in 3′UTR folding upon ADAR knockdown was revealed by PARS-seq analysis and RNA 2D prediction using constraints derived from the PARS-seq experiment (Fig. 3a and Supplementary Fig. 5a).

PSMB2 is also highly edited, with 61 editing sites in its 3′UTR, 21 of them DES, more than in any other gene (Supplementary Datas 8–9). The structural profiles indicate that the 3′UTR structure is affected by ADAR silencing. Fig. 3b illustrates the IR Alu structures at the 3′UTR using the constraints derived from the PARS-seq experiment. Notably, the strong IR Alu structure of PSMB2 3′UTR was shown to affect protein expression^51^; this strong duplex was recently used as ADAR “sponge”^52^, Moreover, the association between PSMB2 3′UTR and ADAR is also supported by the ADAR Clip-seq data we analyzed (Supplementary Table 12). Additional examples are the 3′UTR of MDM4 (93 editing sites, of which 16 are DES), and the 3′UTR of the XIAP gene (87 editing sites, 12 of them are DES) whose structural profiles are also altered by ADAR reduction (Supplementary Fig. 5b, c).

We examined not only the minimum free energy structures but also the structural ensembles predicted using constraints taken from the PARS-seq of control and KD samples. The structural ensembles of IR Alus in F11R and PSMB2 differ significantly between control and KD cells with the KD samples showing fewer DS bases (Wilcoxon test, *p* < 0.0001 for both, Supplementary Table 13). SeqFold^53^ analysis of these two examples reveal the same trend, as in both PSMB2 and F11R the structures are less stable in KD conditions (ΔG control < ΔG KD).

### 3′UTRs adopt different 2D structure upon editing

**Fig. 4:**
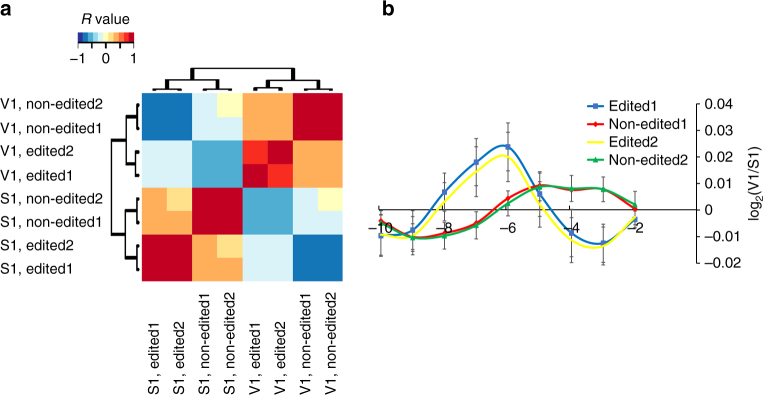
Structural differences between edited molecules and their non-edited versions based solely on control samples data. **a** Heatmap for the correlation between edited and non-edited control samples in a [−50, 50] flanking regions centered on the edited adenosine. Each data point in the vectors being correlated is the number of reads that start in each position over all edited regions. Red: Spearman’s *R* = 1. Blue: Spearman’s *R* = −1. Note that edited reads show higher correlation to edited reads from the other sample and lower correlation to non-edited reads from the same sample. **b** Average relative PARS score for edited and non-edited reads in a region of 10 bases upstream to the edited adenosine. The error bars represent standard errors calculated from the relative PARS scores of the studied regions (87 and 223 regions for edited and non-edited versions, respectively). In both **a** and **b** we only analyzed regions centered on the edited adenosine with sufficient coverage in both replicates (>50 read starts in [−50, 50] region)

Comparison of the structural profiles (at single nucleotide resolution) around edited bases (in regions up to 50 bases upstream to the edited adenosine) and the structural profiles of their non-edited version revealed apparent differences in the local 2D structure (Fig. 4a). This particular analysis was based only on the control samples and not on the ADAR KD cells, so the changes can be solely attributed to editing and not to other editing-independent functions of ADAR.

Moreover, when comparing the relative PARS score upstream to the editing site between edited and non-edited reads, different patterns were inferred (Fig. 4b). Edited transcripts tend to adopt more duplex structures in the 5–9 bases upstream to the editing site, while non-edited transcripts tend to have more compact double stranded structures 2–4 bases upstream to the editing site. This analysis was restricted to bases located upstream to the edited adenosine (as both the read start and the edited position need to be on the same specific read in this analysis). Analysis of lymphoblatoid cells data showed similar patterns (Supplementary Table 14).

**Fig. 5:**
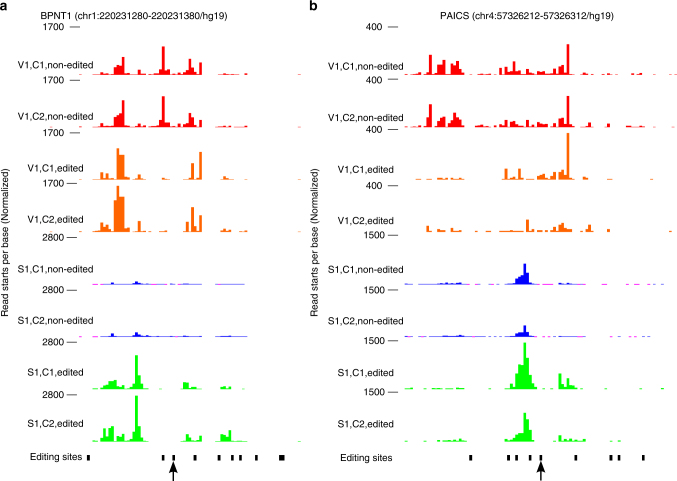
Examples for editing sites associated with differences in 3′UTRs 2D structure between edited and their non-edited versions. **a** Region of [−50, 50] centered at the editing site (chr1:220231330/hg19) in the 3′UTR of BNPT1. **b** Region of [−50, 50] centered at the editing site (chr4:57326262/hg19) in the 3′UTR of PAICS. The editing sites tested in each panel are marked with black arrow. Each track *y*-axis includes the normalized numbers of read starts at each position. Red: V1, non-edited samples, orange: V1, edited samples, blue: S1, non-edited samples, green: S1, edited samples

Some genes show apparent differences in structural profiles in the vicinity of editing sites (50 bases upstream to the edited adenosine) between edited molecules and non-edited molecules (Supplementary Data 11). BPNT1 3′UTR is hyper edited with 37 editing sites, 19 of which are DES. One example for such structurally changed region is shown in Fig. 5a [from −50, to 50 bases] centered at the editing site (chr1:220231330/hg19. CorDiff score = 0.83, empirical *p* < 0.001). BPNT1 3′UTR was also found to be SSC when comparing KD to control samples. Fig. 5b depicts an example for editing associated with SSC (CorDiff score = 0.37, empirical *p* = 0.001) for PAICS 3′UTR in window [from −50, to 50 bases] centered at the editing site in chr4:57326262/hg19. PAICS 3′UTR, which is also hyper-edited, was shown to be involved in nuclear retention^54^.

### RNA editing often stabilizes RNA structures

**Fig. 6:**
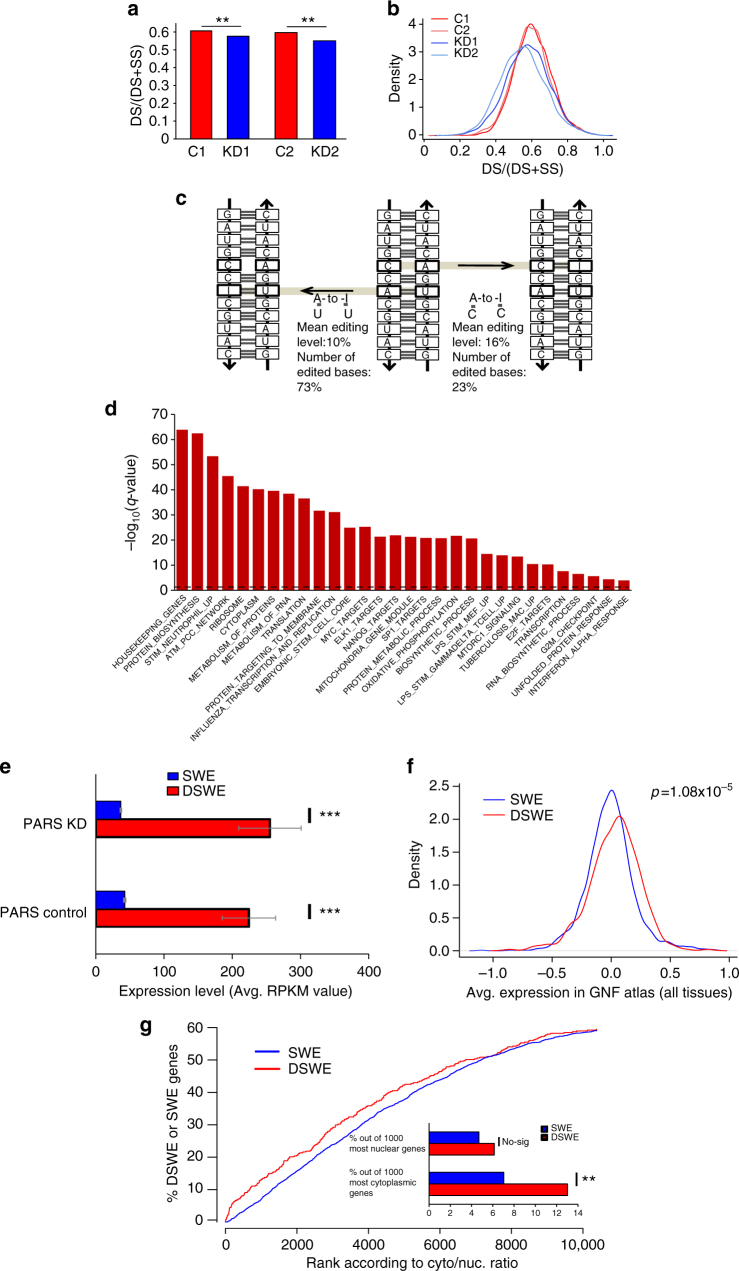
Editing is associated with global changes in 2D structure. **a** The relative number of DS bases in the control samples (red bars) is compared to the relative number of DS bases in the KD samples (blue bars) for each expressed transcript (length > 100 bases and number of read starts > 256). **Wilcoxon test, *p* < 0.01. **b** Density plots are shown for the distribution of per-transcript DS/(DS + SS) values in control and KD. Red: control, replicate 1, pink: control, replicate 2. Blue: KD, replicate 1, light blue: KD, replicate 2. **c** General scheme for editing of A:C and A:U pairs. Editing of A:C into I:C is more frequent than expected by chance (observed: 23%, expected: 13%. Fisher exact test, *p* = 0.001) and its editing level is higher than editing of A:U pairs into I:U. Editing of A:U into I:U occur less than expected by chance (observed: 73%, expected: 82%, Fisher exact test, *p* = 0.006). These numbers are based on alignment of only confident IR Alu duplexes intersected with 3′UTRs and depict the results from Table 2 and Supplementary Table 15. **d** Functional enrichment for DSWE genes. *X*-axis: functional terms. *Y*-axis: –Log_10_(*q*-value). The dashed line indicates *q* = 0.05. **e** Averaged expression level for DSWE (436 genes) and SWE genes (2300 genes) in PARS-seq (averaged from 2 biological replicates per sample in both S1 and V1 treatments, i.e., 4 samples per control and 4 samples per KD). Error bars indicate standard errors. ***Wilcoxon test, *p* < 0.0001. **f** Density plots for the mean expression in DSWE and SWE genes in different tissues based on GNF atlas. Wilcoxon test, *p* = 1.08 × 10^−5^.**g** The percent of DSWE/SWE genes among increasing number of genes ranked by cytoplasmic to nuclear ratio. The gene with the highest cytoplasm/nucleus expression ratio is ranked first, and the one with the lowest cytoplasm/nucleus expression ratio is ranked last. DSWE genes (red line) are enriched within genes highly expressed in the cytoplasm. The inset shows a snapshot of the first 1000 genes with the highest cytoplasmic/nuclear ratio and the lowest cytoplasmic/nuclear ratio. **Fisher exact test, *p* < 0.01

To check how ADAR silencing affects the number of paired (DS) bases, 4021 long and highly expressed transcripts (longer than 100 bases and covered by more than 256 read starts) were subjected to Seqfold pars2spp analysis (PARS to structure preference profile; Ouyang et al.^53^. Pars2spp identifies confident paired (DS) or unpaired (SS) bases in each transcript according to statistical testing of the enrichment of V1 counts and S1 counts, respectively. Using these classifications, we calculated a gene-wise ratio of DS/(DS + SS), and compared it between control and ADAR KD samples. This ratio is found to be significantly lower in the KD samples (Wilcoxon’s test *p* < 10^−15^), namely, the number of confident base pairs identified has been decreased (Fig. 6a, b). A related observation is that edited regions in the control samples are enriched with DS bases compared to the same regions in the KD samples (Supplementary Fig. 6a) and the DS/(DS + SS) ratio is positively correlated with editing level (G/(A + G)) (Supplementary Fig. 6b, Spearman’s *R* ~ 0.12, *p* < 0.01 for both controls).

The first role attributed to ADAR was unwinding of double helical RNA^29, 30^ and it was long assumed that ADAR destabilizes RNA duplexes^55^. This is also consistent with the recent results regarding its role in suppressing innate immune response by destabilizing self dsRNAs^16–19^. However, Wong et al.^25^ found that the preferred substrate for ADAR editing in *GRIA2* site and in human hepatitis delta virus (HDV) is a pseudo-pairing between A and C (A:C) such that deamination of A-to-I, and therefore the formation of I:C pairing, increases RNA stability. We previously^3^ noted that the number of edited A:C pairing (the formation of I:C pairing) is significantly larger than expected by chance, even though in absolute terms most RNA-editing events target A:U pairing. In the current data the number of A:C pairing is also significantly larger than expected by chance (Fisher exact test, *p* = 0.001, Supplementary Table 15). Moreover, we found that editing level at A:C pairing within IR Alus is significantly higher than at A:U sites (averaged-editing level of 16% vs. 10%, for A:C and A:U, respectively. *p* < 10^−15^; Table 2). Recent energy estimations^56^ showed that I:C pairing is about twice as stable as I:U pairing (−5.6 vs. −2.56 kcal per mol, respectively). As depicted schematically in Fig. 6c, we propose that the overall effect of A-to-I editing on DS stability is a trade-off between these two types of base-pair modulations. On the one hand, there is a strong preference to “correct” A:C mismatches (Fig. 6c on the right, with fewer sites but with higher editing level) into more thermodynamically stable I:C pairs. On the other hand, there is an abundant, but statistically less favored, editing of A:U pairs into I:U mismatches^57^ (left part of Fig. 6c, with more sites in absolute numbers but lower editing level). The model shown in Fig. 6c depicts the core results presented in Table 2 and in Supplementary Table 15.

**Table 2:** Average editing level [G/(A + G)] in A:C and A:U pairs within edited IR Alu intersected with 3′UTRs. Editing level [G/(A + G)] is averaged from all control samples

	Min	25%	Median	Mean	75%	Max	St dev	*N*	Wilcoxon’s *p*
A:C pairs	0.0008	0.0316	0.0808	0.1586	0.1888	0.97	0.20	1414	<10^−15^
A:U pairs	0.0004	0.0238	0.0476	0.103	0.125	1	0.14	4378	

We restricted this analysis to sites with base coverage (A + G) > 5 reads

From this model, it is also clear that in the case of perfect DS RNAs, editing can in fact only destabilize the duplex as no A:C mismatches are present. On the other hand, the effect of editing on non-perfect DS RNAs is sequence dependent, and frequently stabilizing.

ADAR1 deficient cells have fewer RNA paired bases, making their mRNAs largely less thermodynamically stable. Interestingly, an interaction between ADAR1 and HuR, a protein that stabilizes mRNA, was recently identified^23^, implying an additional indirect effect of ADAR on RNA stability.

We next examined if the relative number of buried bases (associated with low digestion frequency by both V1 and S1) differs between control and KD samples. Interestingly, transcripts in the KD samples tend to contain relatively more buried bases than transcripts in the control state (Supplementary Table 16. *p* < 10^−15^ for both replicates). Taken together with the above results, we suggest that globally, KD transcripts, which have less defined 2D structures, form a more globular (and less solvent accessible) 3D structure.

### Editing-dependent destabilized transcripts

We further divided the genes into two sets: 1. Genes with higher DS/(DS + SS) ratio in control cells than KD cells, with presumably a stabilizing editing effect, termed “stabilized while edited” (SWE, Supplementary Data 12). 2. Genes with lower DS/(DS + SS) ratio in control cells than in the KD cells, in which editing has presumably destabilizing effect. This set was termed “destabilized while edited” (DSWE, Supplementary Data 12).

We found that the DSWE gene set was significantly enriched for Alu repeats and editing sites over the SWE gene set (Table 3). The DSWE transcripts were also enriched for short windows with identical nearby reverse complementary sequences (Supplementary Table 17). Importantly, we found that our DSWE gene set significantly overlapped (*p* < 0.01) with the gene set of long (>50 bases) hyper-edited duplexes, computed in accordance with the pipeline of Porath et al.^43^ Further analysis of the DSWE gene set revealed a significantly lower free energy of confident IR Alu folding in 3′UTRs (Supplementary Table 18) and higher DS/(DS + SS) ratio in control cells (Supplementary Table 19). Taken together, our finding, that the DSWE gene set is enriched for complementary sequences that fold into highly stable duplexes, is consistent with our observation that A-to-I editing destabilizes this type of secondary structures. As the secondary structures of DSWE transcripts are a priori very stable, editing events which take place will likely reduce their stability. In KD samples the DSWE transcripts are not edited and thus retain more stable RNA duplex regions, (vice versa for SWE transcripts).

**Table 3:** Destabilized while edited (DSWE) genes are frequently edited and enriched with Alu repeats more than stabilized while edited (SWE) genes

	# Editing sites	Summarized genomic size (# bases)	Fold	*p*-value
DSWE genes^a^	4156	14,121,576	1.22	<10^−15^
SWE genes^b^	24,256	100,526,979		
	*# DES*	*Summarized genomic size (# bases)*	*Fold*	*p-value*
DSWE genes	186	14,121,576	1.2	0.0089
SWE genes	1109	100,526,979		
	*# Alu*	*Summarized genomic size (# bases)*	*Fold*	*p-value*
DSWE genes	9587	14,121,576	1.04	6.06 × 10^−10^
SWE genes	65,706	100,526,979		
	*# Alu in IR*	*Summarized genomic size (# bases)*	*Fold*	*p-value*
DSWE genes	7215	14,121,576	1.07	2.54 × 10^−15^
SWE genes	47,881	100,526,979		

^a^Genes that their average ratio of DS/(DS + SS) as resulted from pars2spp analysis (Methods section) is higher in KD than in control samples. Therefore, these genes are suggested to be less stable while edited

^b^Genes that their average ratio of DS/(DS + SS) as resulted from pars2spp analysis (Methods section) is higher in control than in KD samples. Therefore, these genes are suggested to be more stable while edited

To identify the enriched biological processes and molecular functions within the DSWE gene set, we applied Gene Set Enrichment Analysis (GSEA) (Fig. 6d; Supplementary Data 13). Relevant significantly enriched validated gene sets (FDR *q* < 0.05) included housekeeping genes and gene sets involved in a variety of vital biological and metabolic processes, such as protein and RNA biosynthesis. Importantly, enrichment for genes involved in the innate immune response to LPS and type-I interferon response were also detected. Analyses using clusterProfiler (for KEGG pathway enrichment analysis) and GOrilla showed similar functional enrichment signatures.

Liddicoat et al. recently^16^ demonstrated that mouse Adar has an important role in destabilizing dsRNA duplexes formed within a very limited number of mRNA 3′UTRs, by folding of complementary inverted sequences, preventing over-activation of the MDA5-dependent interferon response in the mouse. The enrichment for interferon response genes in the DSWE set hints to a similar mechanism in humans. Interestingly, KEGG pathways analyses revealed that genes involved in systemic lupus erythematosus (SLE) are also enriched. While this result should be cautiously interpreted, as most SLE-related genes in our list are histone genes, over-activation of type-I interferon pathway was observed in both SLE and in AGS^20^. Many symptoms are shared between these pathologies^20^. Our results are also in agreement with the results of Vitali et al.^58^ which found that I:U pairs (which characterize the DSWE genes set) suppress the MDA5-dependent interferon response, while I:C pairs that stabilize the RNA duplex induce the interferon response.

**Fig. 7:**
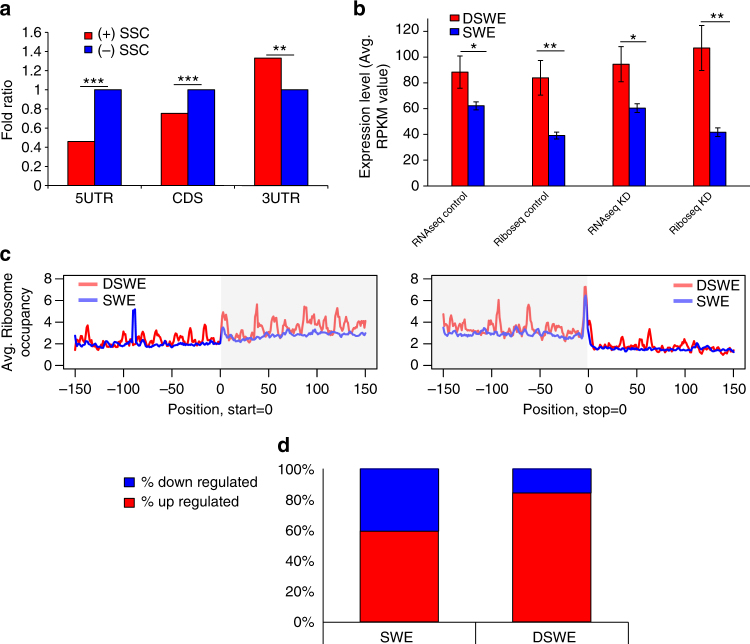
RNA-seq and ribosome profiling analysis. **a** Overlap between secondary structure changed (SSC) exons and the 1000 genes with the highest translation efficiency ratio (Methods section). (+) indicates SSC exons and (−) indicates non-SSC exons (background list). The fold-ratio is calculated according to the overlap with the (−) SSC exons. Only (+) SSC 3′UTR exons are enriched in the 1000 genes with the highest score. Fisher exact test, *p < 0.05, **p < 0.01, ***p < 0.001. **b** Averaged RNA-seq and Ribo-seq expression (averaged from 2 biological replicates of the Ribo-seq) differences (in RPKM values) between DSWE (210 genes) and SWE genes (1196 genes). Error bars indicate standard errors. *t* test, *p < 0.05, **p < 0.01, ***p < 0.001. **c** Mean ribosome occupancy around the start codon (left) and stop codon (right) of the coding sequence (CDS) of DSWE (red) and SWE (blue) genes. CDS is marked with gray shading. KS test *p* < 0.001 for both start/stop codon regions. **d** Percent of upregulated and downregulated genes in the KD samples between DSWE and SWE genes. DSWE genes are more frequently upregulated (Fisher exact test, *p* = 6.67 × 10^−13^)

While analyzing the epigenetic markers of DSWE and SWE genes, a higher signal of H3K4me3 was clearly found for the promoter regions of DSWE, while a higher signal of H3K27me3 was detected in the promoter regions of SWE genes (Supplementary Table 20). In accordance, we found that DSWE genes show a significantly increased expression (~5-fold change; *p* < 10^−15^) compared to SWE genes (Fig. 6e; expression values are available on Supplementary Data 12). A similar result (*p* = 1.08 × 10^−5^) was also obtained by computing relevant GNF microarray data derived from several different tissue types (Fig. 6f). This is also supported by the RNA-seq and ribosome profiling we performed (see below) on control and ADAR KD HepG2 cells (Fig. 7b). Analysis of HepG2 gene expression data from the ENCODE database revealed that DSWE transcripts are enriched in the cytoplasm compared to SWE genes (Fig. 6g). The enrichment of the DSWE set for highly expressed cytoplasmic transcripts is consistent with the possible involvement of these transcripts in response to environmental signals, including the cytosolic immune response to viral nucleic acids. It is also in agreement with the known activity of ADAR p150 in the cytoplasm and its induction during the type-I interferon response^13^.

### 3′UTR structure changes and modulation of translation

Translation is regulated directly by binding of translation factors to the mRNA 3′UTR^59^. This binding may depend on a defined RNA structure. Therefore, to explore if SSC 3′UTRs are associated with protein expression regulation, we conducted RNA-seq and ribosome profiling (Ribo-seq) in control and ADAR1 KD HepG2 cells (Methods section). Genes with SSC in their 3′UTR were found to be enriched for differences between Ribo-seq fold-change (FC) and RNA-seq FC (top 1000 genes in the sorted list of all expressed genes, ranked by their translation efficiency ratio between KD and control; Fig. 7a). The structural changes may be related to modulation of RNA stability and abundance, or to regulation of translation. Interestingly, genes with SSC 3′UTR exons were shown to alter their translation efficiency more than SSC 5′UTR or SSC CDS exons (Fig. 7a), suggesting that ADAR regulation of translated genes is mediated mainly by 3′UTRs.

We found that DSWE are highly translated compared to SWE (Fig. 7b), in agreement with their transcription levels. Ribosome occupancy signatures for DSWE and SWE are significantly different along the transcript (Fig. 7c; KS test *p* < 0.001 for both start/stop codon region), with DSWE genes consistently showing higher signals. More careful inspection of DSWE and SWE translation profile reveals that DSWE are frequently upregulated in the KD samples (Fig. 7d; Fisher exact, *p* = 6.67 × 10^−13^), suggesting that KD of ADAR and reduction of RNA editing may contribute to DSWE mRNA stability and increase DSWE gene translation.

## Discussion

Our experimental data provide insight into the SSC induced by ADAR1 on dsRNA in the entire transcriptome of human cells. We demonstrated that the abundant SSC induced by ADAR1 silencing overlap with A-to-I editing sites in relevant transcripts, implying a direct role for editing in shaping mRNA structure. Yet, it is theoretically possible that some SSC resulted from an indirect editing-independent regulatory function of ADAR1.

RNA bases can easily pair, intra-molecularly, resulting in RNA folding into a variety of secondary structures. Long intramolecular dsRNAs are the major substrate of ADAR1, and the pioneering studies in the RNA-editing field proposed that ADAR1 primarily unwinds dsRNAs^29, 30^. However, our results, rather unexpectedly, show that the effect of editing on the entire transcriptome is to increase the DS/SS ratio. Thus, our data support a modified paradigm whereby RNA editing can enhance or reduce proper intramolecular base pairing in a context-dependent manner. An explanation for the arguably contradicting observations is that in long perfect dsRNA structures, by definition, there are no A:C mismatches, and the only possible effect of editing is to make the structure less stable. However, in the human transcriptome where the dsRNA structures are formed by imperfect Alu repeats, with typically over 15% mismatches, editing that changes A:C mismatches to I:C can contribute to stabilization of the structures.

RNA secondary structure acts as an additional layer of regulation which affects several steps in the gene expression program. The current understanding of the effects of small sequence variations and modifications on RNA structure is however still limited. The current study highlights the potential regulatory role of structural changes that take place following RNA modifications. This understanding is especially relevant in the current era, in which the extent and functional roles of various mRNA modifications, like m^6^A and m^1^A methylation^60, 61^, are being deciphered. Thus, A-to-I editing can play a significant role in this form of regulation as it affects Watson–Crick interactions.

Combined deletion of genes involved in the cytosolic response to dsRNA, *Mda5* and *Mavs*, rescues *Adar* null embryos to birth, suggesting that a major biological function for A-to-I editing of IRs is to prevent abnormal activation of MDA5 followed by a toxic type-I interferon response. While sensing of foreign, infectious agent-derived dsRNAs is essential for proper innate immune response, dampening of immune response to transcripts derived from endogenous repetitive elements (“endovirome”) that fold into long dsRNAs, is essential for the prevention of a deleterious response. We show that RNA editing can mediate both dsRNA stabilization (SWE genes) and destabilization (DSWE genes). The DSWE list of ~400 genes is significantly enriched for potential MDA5 ligands (i.e., complementary inverted Alus that fold into long “perfect” dsRNAs) for which editing of A:U pairs into I:U pseudo-pairs reduces stability. We hypothesize that DSWE transcripts are enriched for long, abundant, “perfect” dsRNA, involved in ADAR1-regulated cross talk with the innate immune system, which in the absence of adequate A-to-I editing may cause an overwhelming activation of MDA5/MAVS dsRNA sensing pathway. Interestingly, DSWE transcripts also contain increased numbers of STAU1 binding sites compare to SWE, suggesting that in DSWE transcript reduced ADAR1 levels may lead to escape from Staufen-mediated-decay and increase translation (as shown here by our Ribo-seq experiment, albeit not statistically significant, one-tail Wilcoxon test, *p* = 0.09).

Our study provides the context to evaluate RNA-editing-dependent SSC throughout the human transcriptome and implies multiple post-transcriptional regulatory roles for this vital function of ADAR1.

## Methods

### Cell line and cell culture

The Human HepG2, hepatocellular carcinoma, cell line (from ATCC) was maintained in Dulbecco’s modified Eagle’s medium, supplemented with 4 mM glutamine, 100 U ml^−1^ penicillin, 100 μg ml^−1^ streptomycin and 10% FBS (all from Thermo Fisher Scientific Inc.). The cells were routinely checked for mycoplasma contamination.

### ADAR1 silencing

HepG2 cells were stably transfected with validated siADAR1 (assay id: 119581, cat. AM51331) or scrambled siRNA (negative control; cat. #AM4635). Both were purchased from Applied Biosystems. The optimal concentration of siRNA was 20 μM. The silencing was performed for 48 h with ~80% silencing levels. Transfections of the indicated siRNAs were performed using a Nucleofector® Device (Amaxa Biosystems, GmbH).

### Western blot analysis and antibodies

Proteins were extracted from cells using RIPA buffer (Sigma-Aldrich) supplemented with protease inhibitor (Roche). Following separation on a SDS-PAGE, proteins were transferred to a nitrocellulose membrane followed by staining with a primary antibody overnight at 40 °C and washed and incubated with the appropriate secondary antibody for 1 h at room temperature. Specific reactive bands were detected using the SuperSignal West Pico Chemiluminescent Substrate (Thermo Scientific). The antibodies used were as follows: anti-ADAR1 (Santa Cruz: sc-73408), anti-actin (Abcam: Ab156302). For ADAR1, the antibody was diluted to 1:2000. For Actin, the antibody was diluted to 1:10,000. The uncropped western blot gel is found in Supplementary Fig. 7.

### Probing mRNA with S1 nuclease and RNAse V1

PARS-seq samples were digested using RNase V1 and S1 nuclease independently and the cleavage sites are captured by adapter ligation followed by high-throughput sequencing. Combing structural information obtained from these two enzymes independently creates greater confidence for whether a base is single or double-stranded than used alone. Here, we used PARS-seq to probe RNA structural changes that occur under ADAR1 silencing conditions of HepG2 cells. In brief, 2ug of poly-A selected mRNA was heated at 90 °C for 2 min and then immediately placed on ice for 2 min. Next 10× RNA structure buffer was added to the tubes that were transferred to a thermal cycler where the temperature was slowly increases from 4 °C to 23 °C over 20 min. These steps ensure that the RNA is properly renaturated and folded under in vitro conditions. For the enzymatic cleavage, each sample tube was either cleaved by RNase V1 or S1 at 23 °C for additional 15 min resulting in 5′P overhangs. The enzymatic reaction was inactivated by phenol:chloroform:isoamyl alcohol, EtoH precipitated and resuspended in DDW for further fragmentation of the RNA into 200bases fragments (fragmentation was done by adding 1× alkaline hydrolysis buffer to the S1 or V1 cleaved RNA at 95 °C for 3 min). Size selection of RNA was done by gel electrophoresis and was followed by 5′ adapter ligation. This size selection step removes very short fragments of RNA, generated from the fragmentation step that could ligate to adapters. Fragmentation products with 3′P groups are converted to 3′OH groups by Antarctic phosphatase, enabling these products to be ligated to 3′ adapters. This step is followed by RT, size selection and PCR to produce a cDNA library that is suitable for high-throughput sequencing.

### Reverse transcription and real-time qPCR

Total RNA from HepG2 cells was isolated using Trizol (Invitrogen) and treated with DNase I (Invitrogen). Poly-A mRNA was isolated using mRNA Sequencing Sample Preparation kit (Illumina Proprietary). Random-primed cDNA synthesis was done on 2 μg of total RNA using M-MLV RT (Invitrogen). Quantitative real-time PCR (qPCR) was performed using a Fast SYBR Green Master mix (Applied Biosystems, 4385612), and qPCR machine ABI 7900HT genetic analyzer (Applied Biosystems) with standard qPCR parameters to analyze the expression of indicated genes compared to the control gene ABL. Results were analyzed by the SDS 2.3 software with the comparative CT method and log10 (Relative quantification values values). Primers and probes sequences are detailed in Supplementary Table 21.

### Libraries preparation and sequencing

Libraries were prepared following the PARS-seq protocol^62^ (briefly described below), using the TruSeq Small RNA sample preparation kit of the Illumina. We ligated the 5′ adapter as described in the TruSeq Small RNA sample preparation protocol (Illumina) and stopped the reaction with 1 µl stop solution (a component of the TruSeq Small RNA sample preparation kit) in 28 C for 15 min. 3′ end treatment with Antarctic phosphatase was done as described in the directional mRNA-Seq sample preparation protocol, followed by its inactivation with ethanol precipitation. 3′ adapter ligation, RT and PCR amplification were performed as described in the TruSeq Small RNA sample preparation protocol. The cDNA was size selected and cleaned using E-Gel 4% agarose (Invitrogen) and Agencourt AMPure XP beads (Beckman Coulter). The library was quantified using Qubit (Invitrogen) and validated using Agilent 2100 Bioanalyzer. Libraries were sequenced on Illumina Hiseq2500 machine. Sequencing yields of the samples are detailed in Supplementary Table 1.

### Short read alignment and read starts counting

Reads were aligned to the human reference genome (hg19) using bowtie2^63^ in local alignment option (bowtie2 -p 6 –local -x hg19_genome_index -U PARS_sample.fastq -S PARS_sample.sam). Only uniquely aligned reads were retained for further analysis. To count the number of reads that start at certain position in the genome we considered only reads that their 5′ matched the reference by at least five bases without gaps, carefully paying attention to the read orientation as PARS-seq protocol is strand specific. Samples were normalized such that at the end of the normalization step the sums of all read starts in each sample were equal. bigWig files containing the results of this counting step were uploaded to UCSC genome browser as custom tracks, for visualization proposes and deposited to GEO database under GEO ID: GSE100210. In order to calculate RPKM values for each gene in the PARS-seq samples, coverage of each gene was calculated using coverageBed of Bedtools package^64^ with RefSeq transcripts annotation^65^ (coverageBed -split -count -abam BAM_FILE.bam -b REFSEQ.bed). The table with RPKM values can be found on Supplementary Data 14.

### Calculation of PARS score for individual bases

We used the definition of Kertesz et al.^32^

1 $${{\rm PARS}\, {{\rm score}}}_{ij} = {\mathrm{Log}}_2\left( {{\mathrm{V}}1_{ij}/{\mathrm{S}}1_{ij}} \right)$$

where V1_*ij*_ and S1_*ij*_ are the normalized count of V1 and S1 read start at base (*j*) in transcript (*i*), respectively. PARS scores for the control cells in this study were also applied for the analysis in Dominissini et al.^61^.

### Calculation of correlation difference between structural profiles

We defined the correlation difference (CorDiff) as follows:

2 $${\mathrm{CorDiff = }}\left( {{\mathrm{Cor}}_{{\mathrm{c1,c2}}}} \right)^{\mathrm{2}}{\mathrm{ + }}\left( {{\mathrm{Cor}}_{{\mathrm{kd1,kd2}}}} \right)^{\mathrm{2}}{\mathrm{-}}\left( {{\mathrm{Cor}}_{{\mathrm{c1,kd1}}}} \right)^{\mathrm{2}}{\mathrm{-}}\left( {{\mathrm{Cor}}_{{\mathrm{c2,kd2}}}} \right)^{\mathrm{2}}$$

where Cor_*ij*_ is the Pearson correlation between sample (*i*) to sample (*j*). c_*i*_ stands for control sample *i* and kd_*i*_ stands for ADAR knockdown sample *i*. Genes and genomic regions for which both CorDiff (V1) > 0 and CorDiff (S1) > 0 were considered as containing SSC regions. In order to calculate empirical *p*-value for this index, we calculated CorDiff based on random shuffle of the reads starts in each region (gene or exon). These CorDiff values were compared with the original CorDiff. Only original CorDiff that was higher than 95% of the random trials (100 random trials for exon level and gene level) was considered as significant (empirical *p* < 0.05).

### Functional enrichment analysis and additional annotations

Functional enrichment analysis of SSC exons was done with DAVID^45^ and GOrilla^46^ using a background list of all exons with a similar coverage. Functional enrichment for the DSWE set was done using the GSEA online web site v6.1, available at: http://software.broadinstitute.org/gsea/msigdb/annotate.jsp, employing the major gene sets collections of the MSigDB (Broad Institute, Massachusetts Institute of Technology). GOrilla and clusterProfiler^66^ were also applied with a background list of all transcripts longer than 100 bases with read starts coverage >256. miR binding sites were taken from TargetScan^67^. STAU1 hiClip data was downloaded from Sugimoto et al.^68^. ADAR1 Clip-seq data was downloaded from Bahn et al.^47^. Histone modification data of HepG2 cells was downloaded from the ENCODE project^69^. Expression level of nuclear and cytoplasmic fractions in HepG2 cells were downloaded from the ENCODE project.

### Defining significant DS or SS bases

Illumina Hiseq2500 sequence reads were aligned to the knownCanonical transcripts list (downloaded from the UCSC table browser), using bowtie2 with local alignment option (bowtie2 -p 6 –local -x knownCanonical.index -U PARS_sample.fastq -S PARS_sample.sam). We only used uniquely aligned reads that their 5′ matched with no gaps for at least five bases (considering the read orientation). Read starts at each position of the transcriptome were counted. Samples were normalized such that at the end of the normalization step the sum of all read starts in each sample was equal. To classify bases as DS or SS or undetermined class (typically low coverage regions), we used the pars2spp script of SeqFold package^53^. This script gets the counts of read starts at each position and outputs the secondary structure class (DS or SS), employing Fisher exact test to assess significance. We ran this procedure on all transcripts and compared the relative number of DS bases in control and KD samples.

### Differential expression and alternative splicing

Differential expression analysis was done using DESeq^70^. Genes with FDR < 0.05 where considered as differentially expressed. Alternative splicing analysis was done using DEXSeq^50^. Exons with FDR < 0.1 were considered as alternatively spliced exons. To eliminate the option that the FDR < 0.1 is too stringent for our purposes (and more SSC exons represent in fact alternative splicing events), we also considered exons with more permissive filter of *p* < 0.05.

To further assess the validity of our PARS-seq method following ADAR1 silencing, a differential gene expression analysis was also performed based on the PARS-seq gene coverage. We found that 2412 and 2626 transcripts were significantly differentially expressed in S1 and V1 samples, respectively, based on DESeq statistics. 1884 of them overlapped (~8-fold enrichment; *p* < 10^−15^ by Fisher’s exact test), increasing our confidence in the data and procedures.

Based on gene FC between KD and control, high correlation between RNA-seq and PARS-seq was found (*R* = 0.67, *p* < 10^−15^).

### RNA-seq and Ribo-seq analysis

RNA-seq and ribosome profiling^71^ (Ribo-seq) reads were aligned to the human reference genome (hg19) using bowtie2^63^ with local alignment option (bowtie2 -p 6–local). Only uniquely mapped reads were retained for further analyses. Coverage of each gene was calculated with coverageBed of Bedtools package^64^ using RefSeq transcripts annotation (coverageBed -split -count -abam BAM_FILE.bam -b REFSEQ.bed) downloaded from UCSC table browser. Using these gene coverage values RPKM values for RNA-seq and Ribo-seq were calculated (can be found on Supplementary Data 14).

For the calculation of translation efficiency (TE)^71^ using Ribo-seq and RNA-seq, only genes with coverage of more than 100 reads in both Ribo-seq and RNA-seq were considered. The FC between ADAR KD and control was calculated using both the Ribo-seq and RNA-seq data with DESeq^70^. TE ratio (TER) was calculated based on the ratio of both FCs:

3 $${\mathrm{FC = Normalized}}\,{\mathrm{read}}\,{\mathrm{count}}\,\left( {{\mathrm{KD}}} \right){\mathrm{/Normalized}}\,{\mathrm{read}}\,{\mathrm{count}}\,\left( {{\mathrm{control}}} \right)$$

4 $${\mathrm{TE = Normalized}}\,{\mathrm{read}}\,{\mathrm{count}}\,\left( {{\mathrm{Ribo {\hbox{-}} seq}}} \right){\mathrm{/Normalized}}\,{\mathrm{read}}\,{\mathrm{count}}\,\left( {{\mathrm{RNA - seq}}} \right)$$

5 $${\mathrm{TER = TE}}_{{\mathrm{KD}}}{\mathrm{/TE}}_{{\mathrm{control}}}{\mathrm{ = FC}}_{{\mathrm{Ribo - seq}}}{\mathrm{/FC}}_{{\mathrm{RNA - seq}}}$$

Ribosome occupancy was calculated using the GWIPS-viz method in Ribogalaxy^72^ Meta-gene analysis was done in the span of 150 bases upstream/downstream to translation start/stop sites.

### RNA-editing detection

Known RNA-editing sites were examined in the bowtie2 alignment files based on list of editing sites gathered from RADAR^8^ and DANRED^42^. This list was used as input for mpileup of samtools package^73^ for detection of variants in defined positions. The mpileup file was parsed using mpileup2snp of VarScan^74^. Changes from A-to-G were retained.

De novo RNA-editing sites in hyper-edited regions were detected using Porath et al.^43^ pipeline. We identified dense editing clusters of high-quality (Phred ≥ 30) A-to-G mismatches, in which the number of A-to-G mismatches was ≥5% of the read length and > 90% of the total number of mismatches. In order to find the editing level of these hyper-edited sites we used the bowtie2 alignment (uniquely aligned reads that were also used for the PARS-seq analysis), and the hyper-edited sites list was served as input for the mpileup–VarScan pipeline.

Known and de novo sites for which their editing level G/(A + G) is significantly lower (*p* < 0.05) in ADAR KD cells were considered as differential-editing sites (DES).

The lists of editing sites are given in Supplementary Datas 1–3.

The list of known RNA-editing sites from RADAR^8^ and DARNED^42^ was also used for editing analysis of lymphoblastoid cells PARS-seq^27^ in a similar manner.

### Analysis of PARS-seq from lymphoblastoid cells

Normalized read start count of V1 and S1 treatments for the samples from Wan et al.^27^ (NA12878 and NA12891, respectively) done on human lymphobalsotid cells were downloaded from GEO (GEO ID: GSE50676). Raw reads (fastq files) for these samples were downloaded from SRA (SRA ID: SRP029656). Mapping, PARS-analysis and editing sites calling were performed as detailed above.

### Prediction of RNA 2D structure and free energy

RNA 2D predictions were performed for selected cases (as in the cases of IR Alu regions of PSMB2 and F11R).

Average PARS scores from both biological replicates were calculated from all bases of the target regions. Bases with PARS score >2.5 were defined as DS and bases with PARS score <−2.5 were defined as SS. Bases with no significant trend were registered as unknown. The binary classifications were then used as constrains for RNAfold^75^ (RNAfold –C) of the Vienna package^75^. In addition, editing sites which were significantly changed between control and KD (defined as DES by previous steps), covered by a total of more than 100 reads with editing level G/(A + G) > 0.1 on average in the control samples, were changed from A-to-G prior to modeling.

Visualization of 2D RNA structures were done using RNAplot of Vienna package^75^. R-chie^76^ was used to draw the arc diagrams of the RNA 2D structures (as in Fig. 3).

In order to compare structural ensembles in control and KD samples, we used RNAsubopt of Vienna package with 1000 random suboptimal structures using constrains derived from our PARS-seq experiment (RNAsubopt –p 1000 –C).

Selected examples (e.g., the F11R and PSMB2 IR Alu regions in Fig. 3) were analyzed additionally by SeqFold.

### Comparison of PARS-seq signal to known RNA structures

PARS score was calculated for each of the RNA molecules shown in Fig. 2 and Supplementary Fig. 1. Solved 3D structures were visualized using Jmol (www.jmol.org) and colored by their PARS scores. Solved 3D structures were assigned to 2D structure using X3DNA/DSSR program^77^ (this was used in Supplementary Fig. 1). Known 2D structures were visualized using VARNA^78^ and colored by their PARS score.

In order to get the correlation heatmap in single-nucleotide resolution as in Fig. 2d, we first chose the 1000 genes with the highest coverage among PARS-seq samples. Next, we filtered for bases were read starts per base in all 8 PARS-seq samples together is >17 reads (>2.125 read starts per base for each sample, on average). Next, we calculated the spearman correlation and plotted the resulted matrix.

### Comparing PARS-seq signal between non-edited and edited reads

Read starts information from reads containing the edited adenosine in control samples was registered. Similar information was register for reads covering the same site containing the non-edited version. Edited and non-edited profiles in control samples were compared using the CorDiff index (see above). Only regions with CorDiff >0 in both V1 and S1 for which the original CorDiff is higher than 95% of the random trials were considered as structurally changed between edited and non-edited versions (as in Supplementary Data 11). In order to draw the structural signature upstream to the edited adenosine, reads starts were normalized to the total read starts in that region. We averaged this normalized count and used it in order to calculate the relative PARS score in the upstream region to the edited adenosine.

### Statistics

All statistical tests were done using R^79^.

### Code availability

All R and Perl scripts used for the different bioinformatics assays can be obtained from the authors upon request.

### Data availability

The datasets generated during the current study are available in the GEO (https://www.ncbi.nlm.nih.gov/geo/) repository, under GEO ID: GSE100210. All raw sequencing data (fastq files) from this study were uploaded to GEO under GEO ID: GSE100210. This includes also processed data of reads starts per base in genome coordinates (hg19, bigwig files) and in transcriptome coordinates (tab-delimited files). All PARS-seq alignment files (in BAM format, aligned to hg19 or to UCSC knownCanonical transcripts) can be downloaded from the following link: http://bioinfo.lnx.biu.ac.il/downloads/Solomon_Paper/

## Electronic supplementary material

Supplementary Information

Description of Additional Supplementary Files

Supplementary Data 1

Supplementary Data 2

Supplementary Data 3

Supplementary Data 4

Supplementary Data 5

Supplementary Data 6

Supplementary Data 7

Supplementary Data 8

Supplementary Data 9

Supplementary Data 10

Supplementary Data 11

Supplementary Data 12

Supplementary Data 13

Supplementary Data 14
