# Supplementary material for: RNA editing by ADAR1 leads to context-dependent transcriptome-wide changes in RNA secondary structure
Source: Nat Commun. 2017 Nov 13;8:1440. doi: 10.1038/s41467-017-01458-8 (PMC5682290; doi:10.1038/s41467-017-01458-8)
Supplement: Supplementary file 1 — Supplementary Information [file 41467_2017_1458_MOESM1_ESM.pdf]

Supplementary Figures

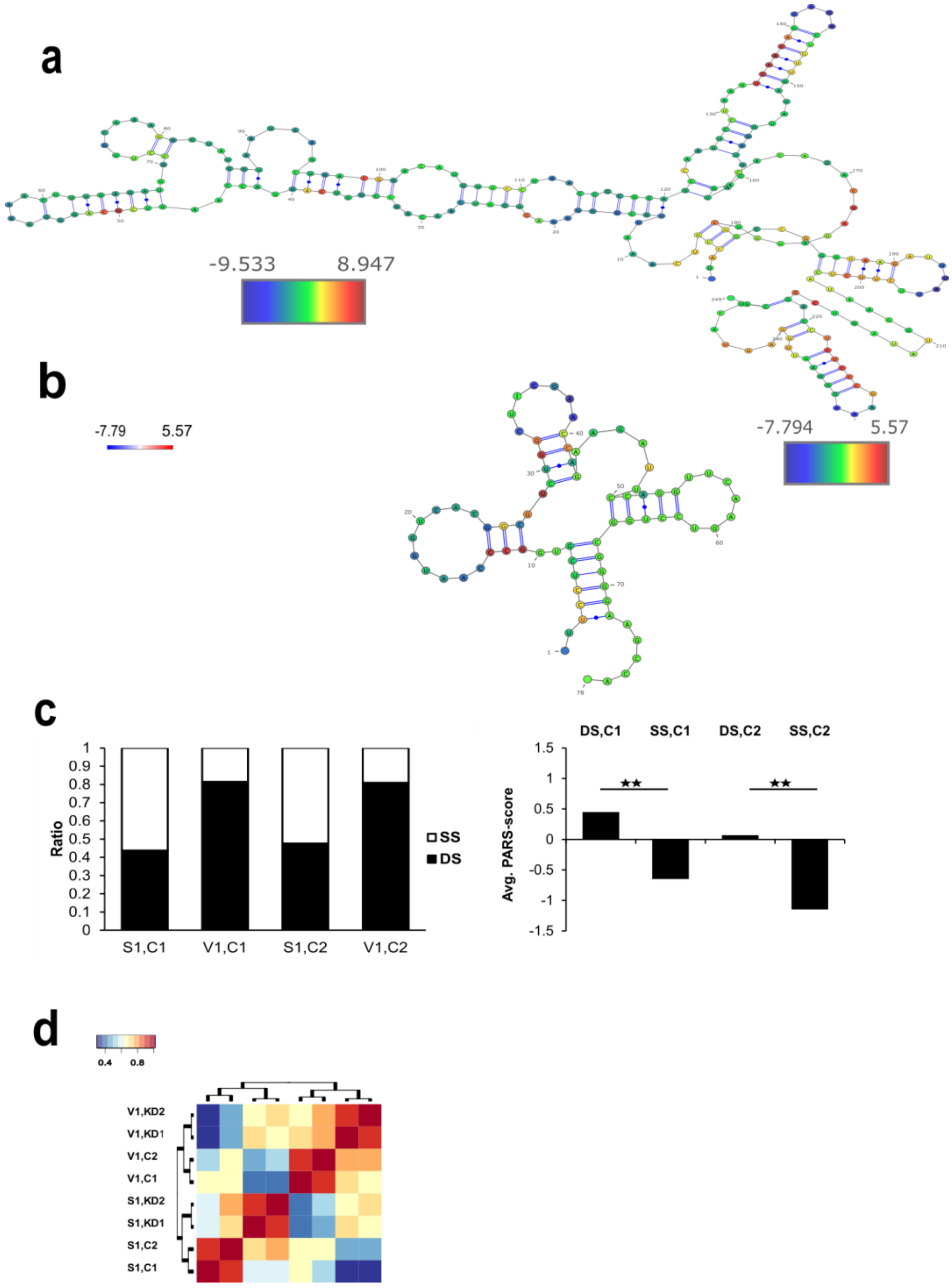

Supplementary Figure 1. PARS-seq verification. **a**. *Tetrahymena* ribozyme 3D solved structure (PDB ID: 1x8w) was reduced to 2D representation by x3DNA/DSSR program (see Methods). The 2D structure was then colored by the PARS-score. Blue – PARS-score < 0 (SS region). Red – PARS-score >0 (DS region). **b.** The solved

structure of tRNA-ARG homolog in yeast (PDB ID: 1f7u) is colored by the PARS-score. **Left** –3D structure is colored by the PARS-score. **Right** – 3D to 2D reduction. The 2D structure is colored by the resulted PARS-score. Blue – PARS-score < 0 (SS region). Red – PARS-score >0 (DS region). **c. Left** – breakdown of read starts at known SS or DS bases based on the solved structure of tRNA-ARG (PDB ID: 1f7u). White – fraction of bases which are known as SS bases. Black – fraction of bases which are known as DS bases. S1, C1 – control 1 cleaved by S1. V1, C1 – control 1 cleaved by V1. S1, C2 – control 2 cleaved by S1. V1, C2 – control 2 cleaved by V1. **Right** – Mean PARS-score. SS, C1 – bases known as SS in control 1. DS, C1 – bases known as DS in control 1. SS, C2 - bases known as SS in control 2. DS, C2 – bases known as DS in control 2. ** p<0.01. **d**. Heatmap for the correlation between samples in the 1000 most highly expressed genes. Spearman correlations were calculated between vectors which include the total read coverage in each gene. (correlations are based on gene level resolution data). Red – Spearman's R=1. blue – Spearman's R=0.2.

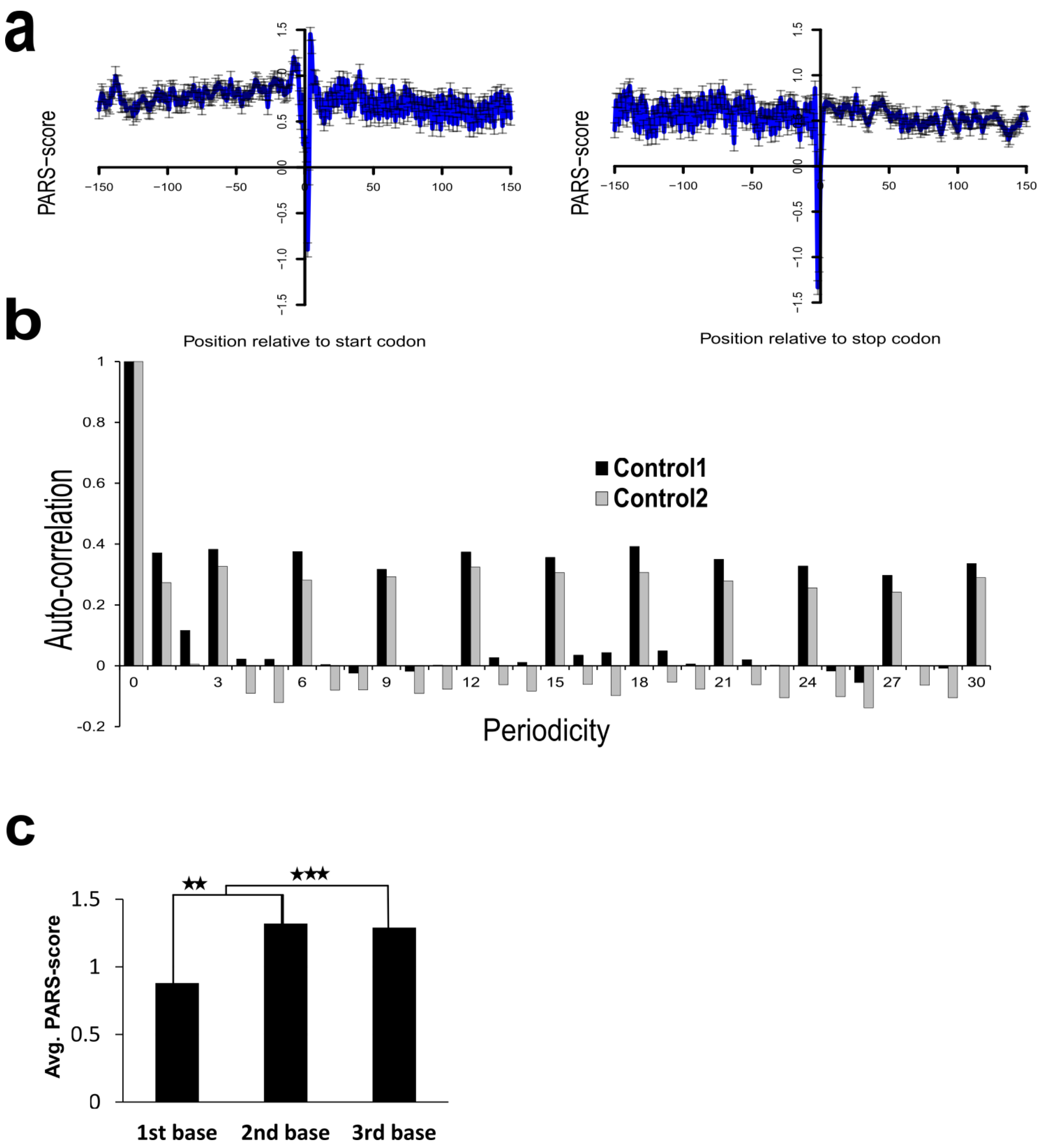

Supplementary Figure 2. PARS-score signatures at CDS. **a.** PARS-score profile of region near the start codon (left) or stop codon (right). Data from both replicates were averaged. Error bars indicate standard errors for each nucleotide using 1244 genes with sufficient coverage in all control samples. **b.** PARS-score periodicity of the CDS. PARS-scores were calculated in region of 150 bases downstream to the start or 150 bases upstream to the end and averaged over all transcripts. The periodicity was measured by auto-correlation. **c.** Average PARS-score in CDS region according to positions in codons. The first base in the codon is significantly less structured than the next two bases. ** - *p* <0.01, *** p<0.001.

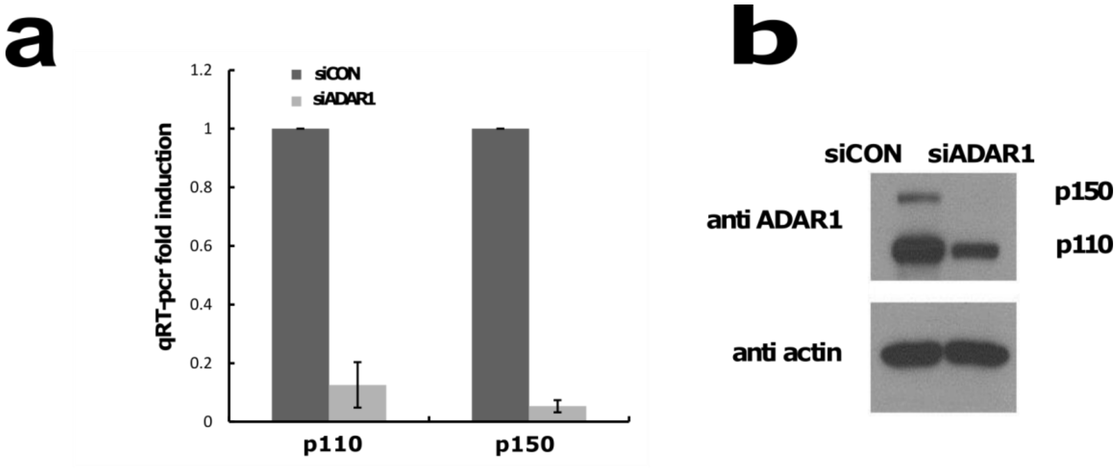

Supplementary Figure 3. HepG2 cells transfection with either siRNA-CON or siRNA-ADAR1. **a.** mRNA expression level of ADAR1-p110 and ADAR1-p150. Normalization was done to the control cells. **b.** Protein expression level of ADAR1. Total cell lysates were immunoblotted with anti-ADAR1 antibodies and with anti-Actin for control.

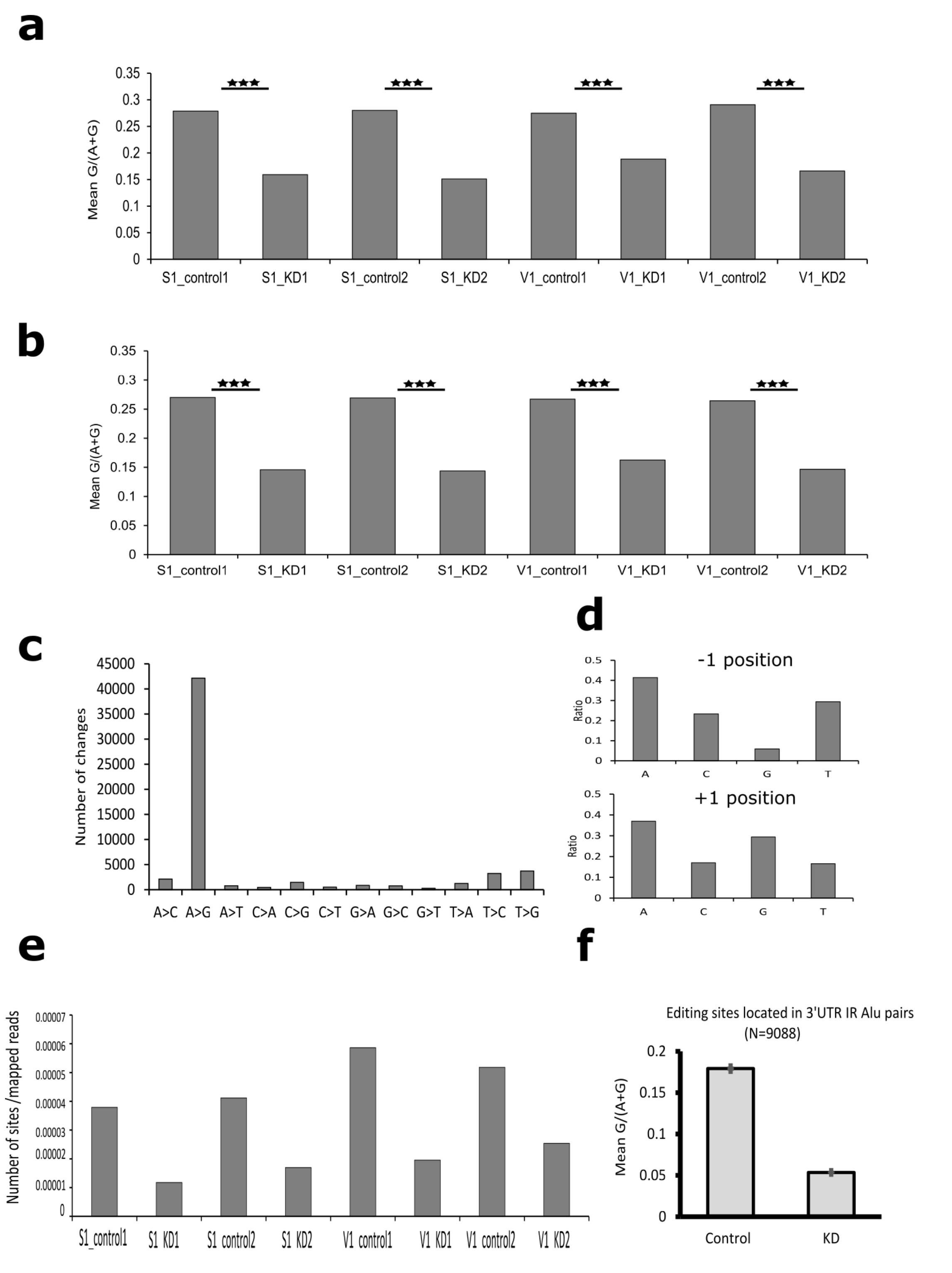

Supplementary Figure 4. Editing levels in the ADAR KD samples. **a.** Mean editing levels for each sample using known editing sites included in DARNED or RADAR. **b.** Mean editing levels for each sample using *de-novo* detection of RNA hyper-editing sites (see Methods). **c.** Abundance of various substitution types in the hyper-editing

detection. **d.** Base compositions around the hyper-edited adenosine. **Top** – (-1) position upstream to the edited adenosine. **Bottom** – (+1) position downstream to the edited adenosine. **e.** Number of hyper-editing sites detected in each sample normalized by the number of mapped reads. **f.** Mean editing level for RNA editing sites located within 3’UTR IR Alus (N=9088 RNA editing sites). *** - $p < 0.0001$.

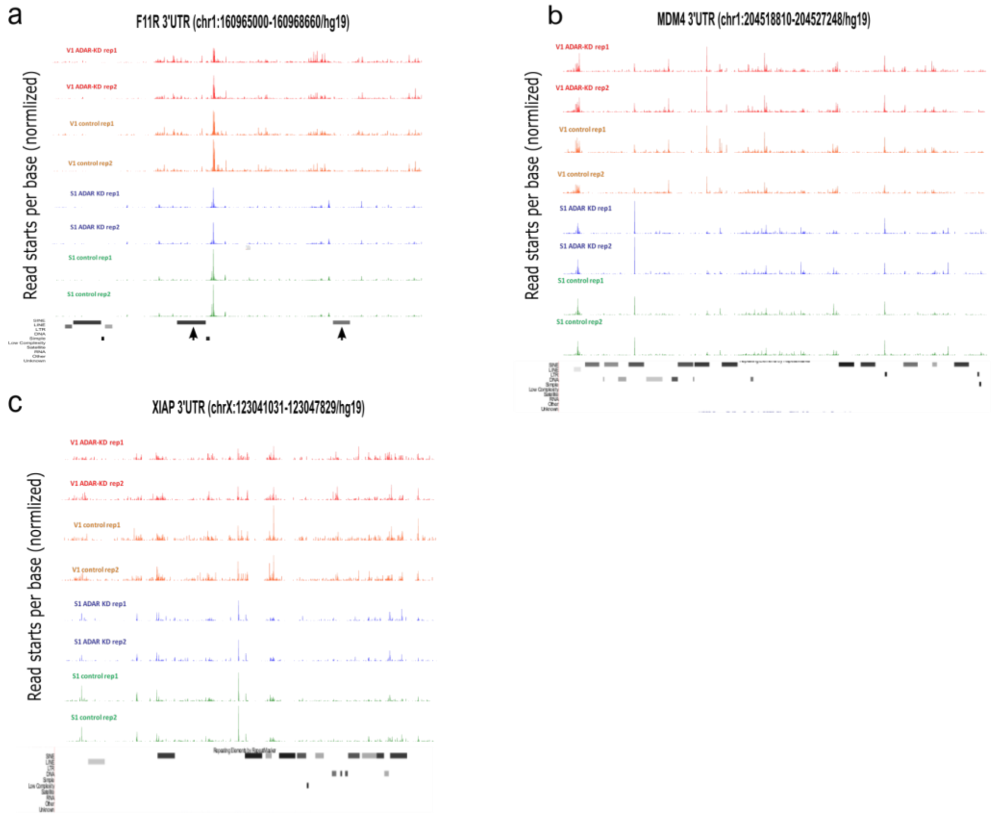

Supplementary Figure 5. Selected cases with apparent structural changes following ADAR silencing. **a.** SSC 3'UTR of F11R (chr1:160966275-160967962/hg19). The modeled IR Alus (in **Fig 3a**) are marked with arrows**.** The average Cordiff between S1 and V1 is 0.0645 (empirical p-value<0.01). **b.** MDM4 3'UTR (chr1:204518810-204527248/hg19). The average Cordiff between S1 and V1 is 0.08795 (empirical p-value<0.01) **c.** XIAP 3'UTR (chrX:123041031-123047829/hg19). The average Cordiff between S1 and V1 is 0.0733 (empirical p-value<0.01) Each track presents the normalized number of read-starts at each position in various samples. Repetitive element (including Alu) are shown at the bottom of each panel and serve as proxy for multiple RNA editing sites.

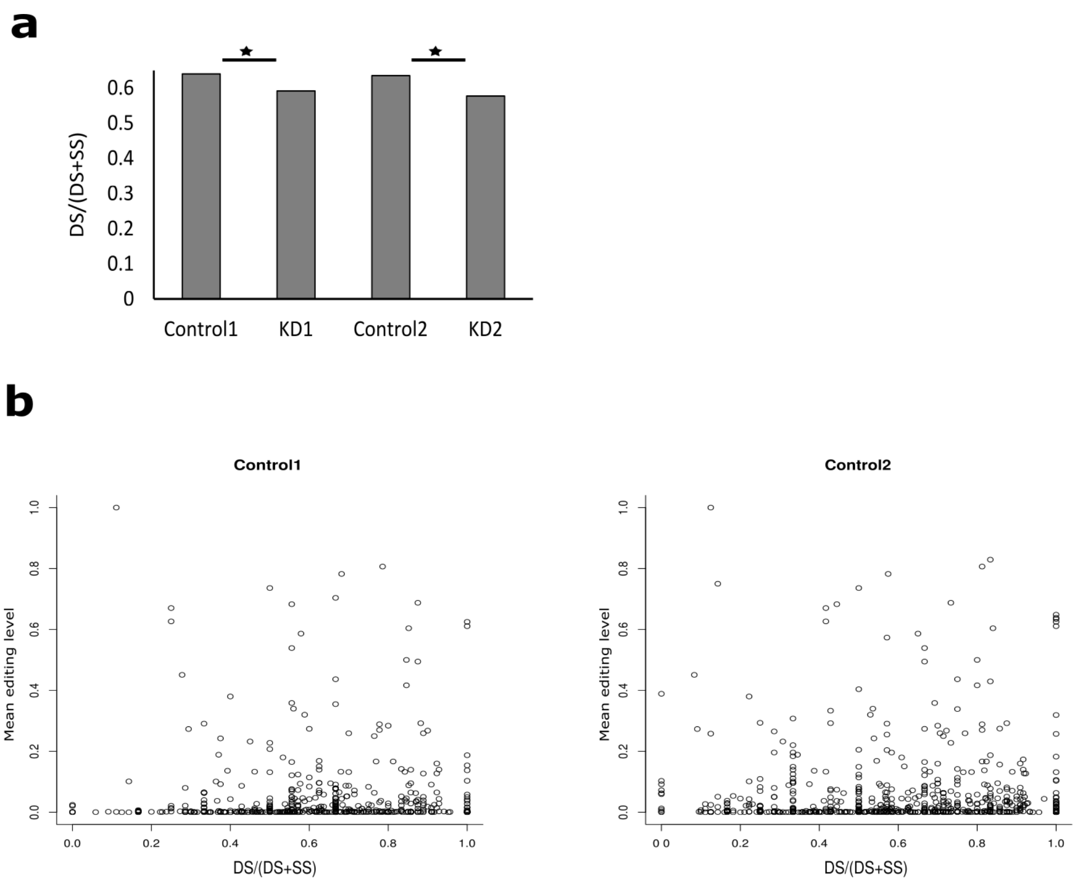

Supplementary Figure 6. Editing level of known editing sites is correlated with RNA 2D structure. **a**. Mean DS/(DS+SS) of edited regions [-50,50] in different samples. * - p <0.05. **b**. Ratio of paired bases DS/(DS+SS) versus editing level. **Left** - control 1. Spearman's R=0.1, p=0.008. **Right -** control 2. Spearman's R=0.135, p=0.0028

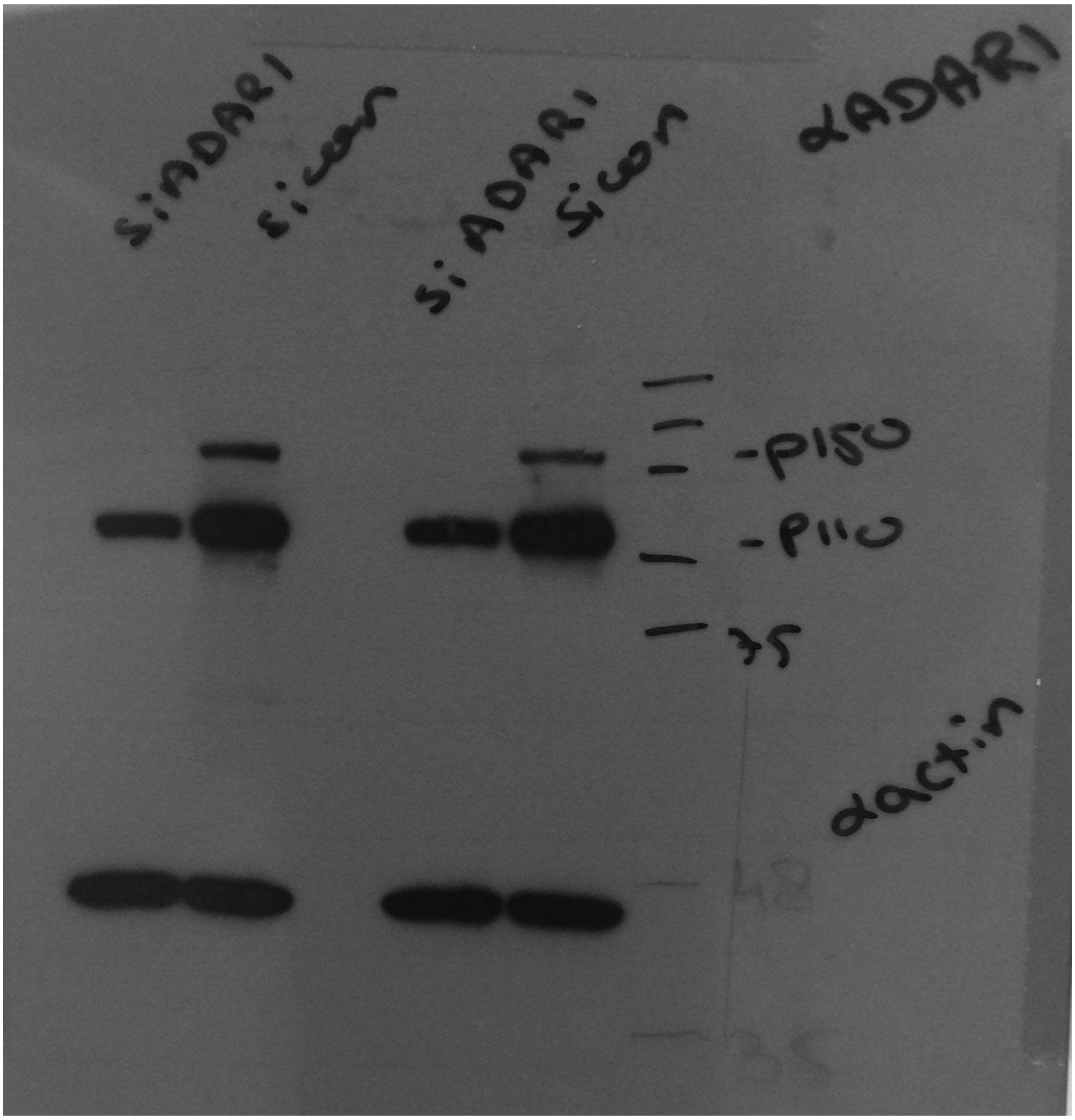

Supplementary Figure 7. The uncropped version of the western-blot presented in Supplementary Figure 3.

Supplementary Tables

Supplementary Table 1. Samples used in this study.

#	Sample ID	Sample Description	S1 or V1	# reads	% reads aligned	# reads uniquely aligned (hg19)
1	SB240	Control	S1	151,786,898	98.64	97,674,173
2	SB241	ADAR-KD	S1	187,281,762	98.58	94,872,193
3	SB242	Control	S1	167,242,064	98.26	96,099,684
4	SB243	ADAR-KD	S1	133,593,141	97.83	69,098,480
5	SB244	Control	V1	151,996,530	97.68	85,167,613
6	SB245	ADAR-KD	V1	291,674,201	97.56	126,700,731
7	SB246	Control	V1	165,991,773	97.12	84,809,964
8	SB247	ADAR-KD	V1	175,935,603	96.97	91,466,868

Supplementary Table 2. Average PARS-score in DS or SS bases of U2 snRNA (UCSC ID: uc021xtz) according to the U2 snRNA conserved RNA structure (RFAM ID: RF00004). Conserved RNA structure was downloaded as Stockholm format file from Rfam.

	**Min**	**25%**	**median**	**mean**	**75%**	**Max**	**std**	**N**	**Wilcoxon test**
SS, Control1	-9.05	-2.93	-1.20	-0.97	0.68	8.89	3.90	21	0.002
DS, Control1	-3.66	1.04	3.49	3.01	4.64	8.37	3.26	14	
SS, Control2	-9.14	-2.63	-0.49	-0.79	0.61	9.15	3.87	21	0.0007
DS, Control2	-2.64	1.67	3.76	3.34	4.72	8.24	3.02	14	

Supplementary Table 3. Structural differences in regions upstream or downstream to start/stop codons as expressed by average PARS-score. C1 – Control sample 1. C2 – Control sample 2.

	Min.	25%	Median	Mean	75%	Max.	Wilcoxon test p-value
C1, Upstream to start	0.27	0.74	0.81	0.81	0.87	1.24	
C1, Downstream to start	-0.89	0.62	0.75	0.72	0.83	1.49	5.78×10^{-6}
C2, Upstream to start	0.22	0.71	0.77	0.77	0.85	1.17	
C2, Downstream to start	-0.91	0.52	0.66	0.63	0.74	1.42	7.58×10^{-14}
C1, Upstream to stop	-1.30	0.48	0.63	0.57	0.72	0.90	
C1, Downstream to stop	-0.11	0.47	0.53	0.53	0.61	0.77	6.72×10^{-5}
C2, Upstream to stop	-1.37	0.48	0.62	0.57	0.71	0.93	
C2, Downstream to stop	-0.04	0.45	0.54	0.53	0.60	0.81	1.06×10^{-5}

Supplementary Table 4. Distribution of PARS-scores in CDS according to base position in the codon. Wilcoxon p-value: in both control1 and control 2: first to second base, first to third base, second to third - < 10^{-15}.

	**min**	**25%**	**mean**	**median**	**75%**	**max**
Control1, first base	-12.92	-1.202	0.96	0.42	2.01	11.99
Control1, second base	-12.65	-1.202	1.32	0.69	2.46	12.13
Control1, third base	-12.9	-1.202	1.32	0.62	2.46	11.54
	**min**	**25%**	**mean**	**median**	**75%**	**max**
Control2, first base	-12.22	-1.31	0.8	0.32	2	11.95
Control2, second base	-12.61	-1.202	1.32	0.61	2.4	11.46
Control2, third base	-12.03	-1.202	1.26	0.53	2.15	12.32

Supplementary Table 5. Alu regions in 3'UTR have significant excess of V1 (DS) reads. The calculation of DS/(DS+SS) is based on the number of bases significantly classified as DS or SS using pars2spp script of SeqFold package[1] (see methods). Only regions with DS+SS > 20 were included in this analysis.

	min	25%	mean	median	75%	max	SD	N	Wilcoxon test
Alu in 3'UTR DS/(DS+SS) in control1	0.109	0.518	0.665	0.633	0.75	0.957	0.182	124	0.0032
3'UTR DS/(DS+SS) in control1	0	0.505	0.607	0.596	0.696	1	0.154	4392	
Alu in 3'UTR DS/(DS+SS) in control2	0.095	0.5	0.633	0.610	0.75	1	0.193	148	0.0001
3'UTR DS/(DS+SS) in control2	0	0.462	0.568	0.560	0.663	1	0.155	5894	

Supplementary Table 6. Lowly covered bases in *Tetrahymena* ribozyme structure are less accessible. PARS-seq reads starts number for each base was compared with getArea result using the solved 3D structure of *Tetrahymena* ribozyme (PDB ID: 1x8w).

	**min**	**25%**	**median**	**mean**	**75%**	**max**	**# bases**	**Wilcoxon test**
Low coverage	9.24	35.64	44.11	51.89	59.33	182.5	89	0.02
High coverage	10.34	39.29	49.24	60.04	65.53	234.2	149	

Supplementary Table 7. The SSC 3'UTR are enriched with Alu, inverted repeat (IR) Alu and ADAR binding sites.

	**# Alu**	**Summarized genomic size (# bases)**	**Fold**	**P-value**
SSC 3'UTR (read starts >50)	528	2274485	1.27	6.00×10^{-10}
Non-SSC 3'UTR (read starts >50)	3564	19443239		
SSC 3'UTR (read starts >200)	377	1550212	1.37	1.27×10^{-8}
Non-SSC 3'UTR (read starts >200)	2901	16311452		
SSC 3'UTR (read starts >1000)	120	433095	1.66	3.9×10^{-8}
Non-SSC 3'UTR (read starts >1000)	1558	9328336		
	**# IR Alu[1]**	**Summarized genomic size (# bases)**	**Fold**	**P-value**
SSC 3'UTR (read starts >50)	897	2274485	1.08	0.00042
Non-SSC 3'UTR (read starts >50)	7125	19443239		
SSC 3'UTR (read starts >200)	617	1550212	1.28	6.75×10^{-9}
Non-SSC 3'UTR (read starts >200)	5060	16311452		
SSC 3'UTR (read starts >1000)	172	433095	1.53	3.63×10^{-8}
Non-SSC 3'UTR (read starts >1000)	2428	9328336		
	**# ADAR binding sites[2]**	**Summarized genomic size (# bases)**	**Fold**	**P-value**
SSC 3'UTR (read starts >50)	87	2274485	1.2	0.044
Non-SSC 3'UTR (read starts >50)	622	19443239		
SSC 3'UTR (read starts >200)	61	1550212	1.17	No-sig
Non-SSC 3'UTR (read starts >200)	550	16311452		
SSC 3'UTR (read starts >1000)	20	433095	1.31	No-sig
Non-SSC 3'UTR (read starts >1000)	328	9328336		

[1] IR alus are all Alu repeats in RepeatMasker table of UCSC that are reversely oriented and within 4000 bases from one another.

[2] ADAR biding sites are based on ADAR CLIP-seq data from Bahn et al. 2015[2] (U87MG cell lines).

Supplementary Table 8. SSC 3'UTR with IR Alus are enriched with STAU1 binding sites.

	**# STAU1 binding sites[1]**	**Summarized genomic size (# bases)**	**Fold**	**P-value**
SSC 3'UTR with IR Alu (read starts >50)	58	227597	1.4	0.0224
Non-SSC 3'UTR with IR Alu (read starts >50)	256	1408445		

[1] Based on STAU1 hiCLIP data from Sugimoto et al. 2015 [3]

Supplementary Table 9. SSC IR Alus in 3'UTRs and overlap with predicted miR binding sites.

	**# miR binding sites[1]**	**Summarized genomic size (# bases)**	**Fold**	**P-value**
SSC IR Alu in 3'UTR (reads starts >50)	31	202150	1.42	0.079
non-SSC IR Alu in 3'UTR (read starts >50)	86	797753		
SSC IR Alu in 3'UTR (reads starts >200)	24	86963	2.07	0.0021
non-SSC IR Alu in 3'UTR (read starts >200)	61	457378		

[1] – Based on predicted miR binding sites from TargetScan[4].

Supplementary Table 10. Number of SSC exons that are included in single-isoform gene compare to multi-isoform genes. The ratios are similar between SSC exons and non-SSC exons.

	**SSC exons[1]**	**Non-SSC exons**	**Total[2]**	**Ratio SSC**	**Ratio non-SSC**	**P-value**
Included in single-isoform gene	407	4384	4791	0.064	0.064	no-sig
Included in multi-isoform gene	5972	64502	70474	0.936	0.936	
Total	6379	68886	75265			

[1] SSC exons are found to be significantly changed in their cleavage pattern between KD and control samples from both V1 and S1; the original CorDiff score (see methods) is higher than 95% of the random trials scores. SSC exons with average number of read starts higher than 50 were tested.

[2] All exons that their average number of reads starts is higher than 50.

Supplementary Table 11. Number of intersections between SSC regions and AS regions. Intersected regions are defined as having overlapping regions longer than 20 bases.

	**Intersected SSC**	**Total SSC[3]**	**Intersected non-SSC**	**Total non-SSC**	**Total intersected**	**Total[4]**	**Ratio SSC**	**Ratio non-SSC**
AS regions[1] (p-value <0.05)	1431	6379	13752	68886	15183	75265	0.2243	0.1996
AS regions[2] (p-value <0.05 and FDR<0.1)	346	6379	3229	68886	3575	75265	0.0542	0.0469

[1] Based on AS analysis by DEXSeq[6]. Regions with p-value <0.05 were considered as AS regions.

[2] Based on AS analysis by DEXSeq[6]. Regions with p-value <0.05 and also FDR<0.1 were considered as AS regions.

[3] Exons are considered as SSC when both V1 and S1 show major differences between KD and control (CorDiff > 0 for both, see methods), the original CorDiff is higher than 95% of the random trials scores (empirical p-value < 0.05). The tested SSC exons have average read starts higher than 50 in all samples.

[4] The tested exons have average read starts higher than 50 in all samples.

Supplementary Table 12. ADAR1 Clip-seq tags (from Bahn et al. 2015) intersected with PSMB2 3'UTR (chr1:36065142-36068867/hg19). Both regions are located within Alu repeats.

chromosome	Start-end (hg19)	strand
Chr1	36066677-36066813	Minus
Chr1	36068156-36068423	Minus

Supplementary Table 13. Structural ensemble results from IR Alu regions in F11R and PSMB2. Using RNAsubopt (-p 1000 -C) tool from RNAfold package [5].

IR Alu	Position (hg19)	Avg. DS/(DS+SS) in Control	Avg. DS/(DS+SS) in KD	Wilcoxon test *p*
PSMB2_IR_Alu_1	chr1:36065451-36066974	0.650	0.641	$<10^{-15}$
PSMB2_IR_Alu_2	chr1:36066658-36067955	0.671	0.666	$<10^{-15}$
F11R_IR_Alu_1	chr1:160965261-160967962	0.641	0.624	$<10^{-15}$
F11R_IR_Alu_2	chr1:160966275-160967962	0.666	0.650	$<10^{-15}$

Supplementary Table 14. Edited molecules have higher DS/(DS+SS) ratio than non-edited molecules. In lymphobalstoid cells from two individuals (Child – NA12879; Father – NA12891; Based on data from Wan et al. 2014 [9]). Changes are not significant.

**DS/(DS+SS)**	**min**	**25%**	**median**	**mean**	**75%**	**max**
Child non-edited	0.069	0.321	0.459	0.463	0.616	0.909
Child edited	0.144	0.364	0.505	0.501	0.643	0.894
Father non-edited	0.117	0.414	0.530	0.537	0.675	0.97
Father edited	0.107	0.405	0.577	0.564	0.715	0.930

Supplementary Table 15. Comparison between edited and non-edited pairs of adenosines in IR Alu within 3'UTRs with only single Alu pair (in order to be sure that this is the legitimate Alu pair). It can be seen that there is an enrichment for A:C editing and depletion of A:U editing compare to background distribution.

	**Edited pairs**	**Non-edited pairs**	**Total pairs**	**Edited ratio**	**Non-edited ratio**	**Fisher test *p***
A:A	5	243	248	0.033	0.017	No sig.
A:C	35	1917	1952	0.23	0.13	0.001
A:G	1	428	429	0.006	0.03	No sig.
A:T	112	11929	12041	0.73	0.82	0.006
Total	153	14517	14670	1	1	

Supplementary Table 16. The distribution of fraction of poorly-covered bases out of the transcript length per each transcript in control and KD samples. Only transcripts with average read start per base >5 for both V1 and S1 were taken for this analysis.

	**min**	**25%**	**median**	**mean**	**75%**	**max**	**stdev**	**N**	**Wilcoxon's *p***
Control1	0.040	0.532	0.637	0.612	0.719	0.977	0.154	1209	$< 10^{-15}$
KD1	0.077	0.596	0.694	0.664	0.757	0.979	0.137	1209	
Control2	0.061	0.523	0.634	0.607	0.712	0.981	0.153	1199	$< 10^{-15}$
KD2	0.086	0.603	0.699	0.670	0.766	0.986	0.143	1199	

Supplementary Table 17. Number of identical reverse complement windows (window size=5 bases) per sequence size for destabilized while edited (DSWE) sequences and for stabilized while edited (SWE) sequences. Analysis was done at the transcript level and at 3'UTR level.

	**min**	**25%**	**median**	**mean**	**75%**	**max**	**stdev**	**N**	**Wilcoxon's p**
DSWE transcripts[1]	5.08×10^{-4}	7.7×10^{-4}	9.47×10^{-4}	0.00116	0.00128	0.00901	7.29×10^{-4}	581	1.65×10^{-10}
SWE transcripts[2]	5.11×10^{-4}	7.18×10^{-4}	8.65×10^{-4}	0.00098	0.00110	0.00933	4.41×10^{-4}	2508	
	**min**	**25%**	**median**	**mean**	**75%**	**max**	**stdev**	**N**	**Wilcoxon's p**
DSWE 3'UTRs[1]	0.00054	0.00124	0.00182	0.00261	0.0033	0.0143	0.00203	505	0.024
SWE 3'UTR[2]	0.00052	0.00117	0.00175	0.0024	0.0029	0.0171	0.00189	1861	

[1] – Transcripts/3'UTRs that their average ratio of DS/(DS+SS) as resulted from pars2spp analysis (see methods) is higher in KD than in control samples. Therefore, these genes are suggested to be less stable while edited.

[2] – Transcripts/3'UTRs that their average ratio of DS/(DS+SS) as resulted from pars2spp analysis (see methods) is higher in control than in KD samples. Therefore, these genes are suggested to be more stable while edited.

Supplementary Table 18. IR Alu in DSWE 3'UTRs show lower predicted ΔG per sequence base. Here, only IR Alu (pair of Alu with opposite orientation with less than 5000 bases between them) within 3'UTRs with only single pair of IR Alus are analyzed. This to be sure that the calculated pair is the legitimate one.

	ΔG per base < (-0.48)	ΔG per base ≥ (-0.48)	Fisher's *p*
IR Alu in DSWE 3'UTRs	6	36	0.03
IR Alu in SWE 3'UTRs	6	145	

Supplementary Table 19. Distribution of DS/(DS+SS) for control samples for DSWE and SWE genes. Values were averaged from both replicates.

	**min**	**25%**	**mean**	**median**	**75%**	**max**	**stdev**	**N**	**Wilcoxon's *p***
DSWE genes	0.076	0.539	0.614	0.611	0.686	0.948	0.120	581	0.048
SWE genes	0.243	0.537	0.601	0.604	0.667	0.974	0.101	2508	

Supplementary Table 20. Distribution of score per base for epigenetic signals intersected with DSWE or SWE promoter regions. Epigenetic data was taken from HepG2 cells in the ENCODE project.

H3k4me3	min	25%	median	mean	75%	max	stdev	N (number of tags)	Wilcoxon's *p*
SWE[1]	1.002	1.883	3.03	3.543	4.651	25.04	2.196	133050	0.003
DSWE[2]	1.002	1.883	3.086	3.594	4.742	21.52	2.219	27622	
H3k27me3	min	25%	median	mean	75%	max	stdev	N (number of tags)	Wilcoxon's *p*
SWE[1]	1.002	1.066	1.163	1.256	1.339	3.014	0.288	5784	0.26
DSWE[2]	1.002	1.063	1.162	1.234	1.322	2.435	0.238	1175	

[1] – Promoter regions of stabilized while edited (SWE) genes.

[2]- Promoter regions of destabilized while edited (DSWE) genes.

Supplementary Table 21. Primers and probes sequences

Gene and orientations	sequence
ADAR110 F[1]	GTGTCCCGAGGAAGTGCAA
ADAR110 R[2]	TGTCTGTGCTCATAGCCTTGAAA
ADAR110 P[3]	TTCCCTCAGCGGATACTACACCCATCC
ADAR150 F	CGGGCGCAATGAATCC
ADAR150 R	TGTGCTCATAGCCTTGAAATGG
ADAR150 P	TTCCCTCAGCGGATACTACACCCA
ABL F	TGGAGATAACACTCTAAGCATAACTAAAGGT
ABL R	GATGTAGTTGCTTGGGACCCA
ABL P	CCATTTTTGGTTTGGGCTTCACACCATT

[1] Forward
[2] Reverse
[3] Probe

Supplementary References

1. Ouyang, Z., Snyder, M.P. & Chang, H.Y. SeqFold: genome-scale reconstruction of RNA secondary structure integrating high-throughput sequencing data. *Genome Res* **23**, 377-87 (2012).
2. Bahn, J.H. et al. Genomic analysis of ADAR1 binding and its involvement in multiple RNA processing pathways. *Nat Commun* **6**, 6355 (2015).
3. Sugimoto, Y. et al. hiCLIP reveals the in vivo atlas of mRNA secondary structures recognized by Staufen 1. *Nature* **519**, 491-4 (2015).
4. Lewis, B.P., Burge, C.B. & Bartel, D.P. Conserved seed pairing, often flanked by adenosines, indicates that thousands of human genes are microRNA targets. *Cell* **120**, 15-20 (2005).
5. Hofacker, I.L. et al. Fast folding and comparison of RNA secondary structures. *Monatshefte für Chemie* **125**, 167–188 (1994).
6. Anders, S., Reyes, A. & Huber, W. Detecting differential usage of exons from RNA-seq data. *Genome Res* **22**, 2008-17 (2012).
7. Sabarinathan, R. et al. The RNAsnp web server: predicting SNP effects on local RNA secondary structure. *Nucleic Acids Res* **41**, W475-9 (2013).
8. Halvorsen, M., Martin, J.S., Broadaway, S. & Laederach, A. Disease-associated mutations that alter the RNA structural ensemble. *PLoS Genet* **6**, e1001074 (2010).
9. Wan, Y. et al. Landscape and variation of RNA secondary structure across the human transcriptome. *Nature* **505**, 706-9 (2014).
