# Supplementary material for: RNA editing by ADAR1 leads to context-dependent transcriptome-wide changes in RNA secondary structure
Source: Nat Commun. 2017 Nov 13;8:1440. doi: 10.1038/s41467-017-01458-8 (PMC5682290; doi:10.1038/s41467-017-01458-8)
Supplement: Supplementary file 2 — Description of Additional Supplementary Files [file 41467_2017_1458_MOESM2_ESM.pdf]

Description of Additional Supplementary Files

File Name: Supplementary Data 1
Description: Editing sites.

File Name: Supplementary Data 2
Description: Differential editing sites (DES).

File Name: Supplementary Data 3
Description: Editing sites that show higher levels in KD.

File Name: Supplementary Data 4
Description: The number of SCC exons in coverage criteria.

File Name: Supplementary Data 5
Description: List of SSC genes with Avg. number of read starts >0.

File Name: Supplementary Data 6
Description: List of SSC exons with Avg. number of read starts >50.

File Name: Supplementary Data 7
Description: GO enrichment from DAVID and Gorilla.

File Name: Supplementary Data 8
Description: Number of editing sites in each SSC 3'UTR.

File Name: Supplementary Data 9
Description: Number of differentially edited sites (DES) in each SSC 3'UTR.

File Name: Supplementary Data 10
Description: Sig. AS regions from DEXSeq (p-value <0.05 and FDR<0.1).

File Name: Supplementary Data 11
Description: Edited regions significantly changed between edited and pre-edited versions.

File Name: Supplementary Data 12
Description: Table of the calculated DS/(DS+SS) ratios for each transcript longer > 100 bases, read starts >256, with both replicates in the same direction and average DS/(DS+SS) difference between KD and control is > 0.02 or <-0.02. Also, RPKM values for the different data sets are included here.

File Name: Supplementary Data 13
Description: Functional enrichment analysis for DSWE genes using GSEA.

File Name: Supplementary Data 14
Description: RPKM values for PARS, RNA-seq and Ribo-seq samples in our study.
